# r*Bm*αTX14 Increases the Life Span and Promotes the Locomotion of *Caenorhabditis Elegans*

**DOI:** 10.1371/journal.pone.0161847

**Published:** 2016-09-09

**Authors:** Lan Chen, Ju Zhang, Jie Xu, Lu Wan, Kaixuan Teng, Jin Xiang, Rui Zhang, Zebo Huang, Yongmei Liu, Wenhua Li, Xin Liu

**Affiliations:** 1 Key Laboratory of Combinatorial Biosynthesis and Drug Discovery, Ministry of Education, and School of Pharmaceutical Sciences, Wuhan University, Wuhan, 430071, China; 2 School of Life Science, Wuhan University, Wuhan, 430071, China; 3 Guangdong Province Key Laboratory for Biotechnology Drug Candidates, School of Biosciences and Biopharmaceutics, Guangdong Pharmaceutical University, Guangzhou, 510006, China; CSIR-Central Drug Research Institute, INDIA

## Abstract

The scorpion has been extensively used in various pharmacological profiles or as food supplies. The exploration of scorpion venom has been reported due to the presence of recombinant peptides. r*Bm*αTX14 is an α-neurotoxin extracted from the venom gland of the East Asian scorpion *Buthus martensii* Karsch and can affect ion channel conductance. Here, we investigated the functions of r*Bmα*TX14 using the *Caenorhabditis elegans* model. Using western blot analysis, r*Bm*αTX14 was shown to be expressed both in the cytoplasm and inclusion bodies in the *E*.*coli Rosetta* (DE3) strain. Circular dichroism spectroscopy analysis demonstrated that purified r*Bm*αTX14 retained its biological structures. Next, feeding nematodes with *E*.*coli Rosetta* (DE3) expressing r*Bm*αTX14 caused extension of the life span and promoted the locomotion of the nematodes. In addition, we identified several genes that play various roles in the life span and locomotion of *C*. *elegans* through microarray analysis and quantitative real-time PCR. Furthermore, if the amino acid site H_15_ of r*Bm*αTX14 was mutated, r*Bm*αTX14 no longer promoted the *C*. *elegans* life span. In conclusion, the results not only demonstrated the functions and mechanism of r*Bm*αTX14 in *C*. *elegans*, but also provided the new sight in the utility of recombinant peptides from scorpion venom.

## Introduction

Scorpions, which are considered ‘living fossils’, maintain primary Paleozoic scorpions features such as the venom apparatus, book lung and pectin [1]. Although Scorpion venom has toxin effects, it also contains enzymes, including hyaluronidase, phospholipase and proteases [2–4], and small peptides with antimicrobial and anti-parasitic activities [5–7]. Scorpions are also used as a source of Chinese medicine to treat stroke and other diseases.

Scorpion venom contains many polypeptides, usually several amino acid residues compiled into long single chains [8–10]. These polypeptides specifically interact with ion channels, causing their blockage or by altering the opening and closing of the channels [11, 12]. Several types of scorpion venom such as chlorotoxin, AaeAP1 and AaeAP2 have anti-cancer effects [13, 14]. Additionally, r*Bm*αTX14, a peptide extracted from East Asian *Buthus martensii* Karsch, is known to be a potent blocker of the Na^+^ currents of root ganglia neuron. The r*Bm*αTX14 cDNA sequence was obtained from the *Bm*K cDNA library [15], and the recombinant protein was successfully expressed both in *Pichia pastoris* and in *E*.*coli* [16, 17].

In this work, we used the animal model *C*. *elegans* to assess r*Bm*αTX14 function. *C*. *elegans* was the first multicellular organism to have its entire genome sequenced, and approximately 35% of *C*. *elegans* genes have human homologs). Additionally, this small soil nematode has a short life cycle [18, 19]. All of these features make *C*. *elegans* a unique model, especially for life span study and disease analysis [20, 21]. In our study, we used the *C*. *elegans* model system to investigate the bioactivity of r*Bm*αTX14, and the results show that this polypeptide plays potential roles against aging and promotes locomotion.

## Materials and Methods

### Plasmid construction

The r*Bmα*TX14 DNA sequence was amplified via the Polymerase Chain Reaction (PCR) with the forward primer CCCATATGGTTCGGGATGCT and the reverse primer CGGGATCCTCAATGGCATTT. After digestion with the *Nde* I and *BamH* I restriction enzymes (TaKaRa, Kyoto, Japan), the r*Bmα*TX14 DNA sequence was ligated into the pET28a vector with T4 DNA ligase (TaKaRa, Kyoto, Japan). The insertion accuracy was verified by DNA sequencing (GENWIZ, Suzhou, China). The antibody was obtained from Proteintech (Wuhan, China).

Since the protein sequence of *Bmα*TX14 was “VRDAYIAKPENCVYHCATNEGCNKLCTDNGAESGYCQWGGKYGNACWCIKLPDDVPIRVPGKCH”, and then the cDNA sequence of the negative control for *Bmα*TX14 was created according to the sequence of *Bma*TX14. The protein sequence of this negative control was “VCHGKPRVPIDVPDKLIWCCNAYGGKWGCQGYEASNGTDLCNKGCNEATHYCNVEPCAKYIDRA”. So the random protein was expressed as the negative control using pET28a-r*Bmα*TX14 (NEG).

### Strains and nematode culture

The *C*. *elegans* strain N2 (wide type) and *E*. *coli* strain OP50 were obtained from the Caenorhabditis Genetics Center at the University of Minnesota. *C*. *elegans* were grown at 20°C on nematode growth media (NGM) plates and were propagated on *E*. *coli* OP50 using standard methods [22]. Synchronization was performed using the standard alkaline hypochlorite method.

### Induced r*Bmα*TX14 expression

The recombinant pET28a-r*Bm*αTX14 plasmid (with 6-His-Tag) or the empty pET28a vector was transformed into competent *E*.*coli* strain *Rosetta* (DE3) cells (Proteintech Group, Wuhan, China). Then, *E*.*coli* cells were maintained at 37°C in Luria–Bertani medium with vigorous shaking. Isopropyl-β-D-thiogalactopyranoside (Amresco, OH, USA) was added at a concentration of 1 mM when the OD_600_ of the *E*.*coli* reached 0.4. After further incubation at 28°C for 4 h, the cells were harvested for further use.

### Western blotting analysis

The *E*.*coli* cells were harvested and lysed with ultrasonication, and the lysate was centrifuged at 4,000 rpm for 10 minutes at 4°C. Then, the supernatant was centrifuged at 12,000 rpm for 15 minutes at 4°C. The supernatant was obtained from the cytoplasm, while the insoluble fraction primarily included inclusion bodies. The supernatant and insoluble fractions were lysed again for 10 minutes at 100°C. Then, the protein samples were analyzed by western blotting.

### HPLC purification

The r*Bmα*TX14 inclusion bodies were lysed in denaturation solution (6 M guanidine-HCl, 0.1 M Tris-HCl, 1 mM EDTA, 30 mM reduced glutathione, pH 8.0). After 2 h of incubation, the solution was slowly added to 100-fold volume of renaturation solution (0.2 M ammonium acetate and 0.2 mM oxidized glutathione, pH 7.0) and incubated at 16°C for 24 h. The precipitate was removed by centrifugation at 12,000 rpm for 15 min. The supernatant was desalted and concentrated with a centrifugal filter device (cutoff value > 3 kDa) at 5,000 g for 4 h. Next, 0.1% TFA was added to the concentrated protein solution to remove the precipitate, and the supernatant was injected into RP-HPLC. Renatured r*Bmα*TX14 was purified by RP-HPLC on a C18 column (10×250 mm, 5 μm) (Elite-HPLC) using a linear gradient of 5–95% acetonitrile with 0.1% TFA in 60 min at a constant flow rate of 5 ml/min, and the protein was detected at 230 nm. The r*Bmα*TX14 peak appeared at 21 min and was manually collected.

### Circular dichroism (CD) spectroscopy

The 20 μM peptide far-UV CD spectra in H_2_O was measured in the 195–250 nm wavelength range (protein secondary structure) at 25°C on a Jasco J-810 spectropolarimeter (Jasco Corporation, Tokyo, Japan) with a 0.1 cm pathlength cylindrical cell. The bandwidth was 1 nm, and the response time was 1 s. All the samples were allowed to equilibrate thermally for 5 min prior to the CD measurements. Each sample spectrum was corrected by subtraction from the spectrum (baseline) that was recorded for H_2_O. Each spectrum is an average of three different scans that were obtained by collecting data at 0.1 nm intervals at a scan speed of 200 nm/min.

### Life span analysis

All life span assays were conducted in 96-well plates using liquid culture at 20°C as previously described [23]. Batches of synchronized L1 nematodes were incubated in S medium containing *E*. *coli* OP50 (initial *D*570 of 0.6–0.7), 50 μg/mL carbenicillin and 0.1 μg/mL fungizone with gentle shaking until L4. Next, 2′-deoxy- 5-fluorouridine (Ribio, Beijing, China) was added to prevent progeny growth. After further incubation for 24 h, culture aliquots were dispensed into plate wells (90 μL/well, with approximately ten adults; five to ten wells/sample), and appropriate volumes of *E*.*coli* solution were added to achieve the indicated concentrations. The total incubation volume was 100 μL per well, and the plates were sealed with Parafilm to prevent evaporation. The day of nematode transfer was counted as day 0 in the life span analysis. The fraction of nematodes alive was scored microscopically every 2 days. Survival data were analyzed using the log-rank test using SPSS 17.0 for Windows.

### Food clearance assay

Newly synchronized L1 nematodes were incubated in 96-well plates containing foods (*E*. *coli* strain r*Bm*αTX14 or the control with initial OD_600_ of 0.6–0.7) with gentle shaking (110 rpm) at 20°C. Regarding the food clearance assay, the plates were measured every day using the microplate spectrophotometer for a week as previously described [24].

### Locomotor behavior assay

Locomotor behavior was assessed including the reversal frequency and body bend as previously described, with a slight modification [25]. Adult nematodes were transferred to the plates lacking food, and the animals were allowed to adjust to the plates for 5 minutes. Video images were recorded and analyzed off-line. Body bend frequency was quantified by counting the number of body bends produced by 20 worms in 30 seconds using a SAMSUNG SCC-101BP device. Reversals of the nematodes were also measured with VideoMach software using 25 nematodes per group.

### Total RNA extraction and microarray analysis

Total RNA was extracted from adult worms grown at 20°C on NGM plates using TRIzol™ Reagent (Invitrogen, Carlsbad, USA) according to the manufacturer’s instructions. We further examined the gene expression changes through DNA microarray expression profiling. Affymetrix Genechips were used to perform *C*. *elegans* Genome Arrays and the samples were tested by CapitalBio Corporation (Beijing, China). The results were analyzed using the Molecule Annotation System (MAS) 3.0 (at http://bioinfo.capitalbio.com/mas3/).

### Quantitative real-time PCR analysis

The mRNA was converted to cDNA using the RevertAid First Strand cDNA Synthesis Kit (Thermo, Waltham, USA) according to the manufacturer’s instructions. *Act-1* expression was used as the control reference. Quantitative RT-PCR was conducted using SYBR Green PCR Master Mix (TOYOBO, Osaka, Japan) and analyzed with the 7500HT Fast Real-Time PCR machine (Applied Biosystems, Waltham, USA).

### Live subject statement

All experiments were performed in compliance with the relevant laws and institutional guidelines, and approved by the Committee of Experimental Animal Administration in the School of Pharmaceutical Sciences, Wuhan University.

### Statistical analysis

The results are expressed as the mean ± S.E. variance. The Tukey-Kramer multiple comparisons test was used to determine statistical significance. A p-value of <0.05 was considered to be statistically significant.

## Results

### r*Bmα*TX14 was expressed both in the cytoplasm and inclusion bodies

**Fig 1:**
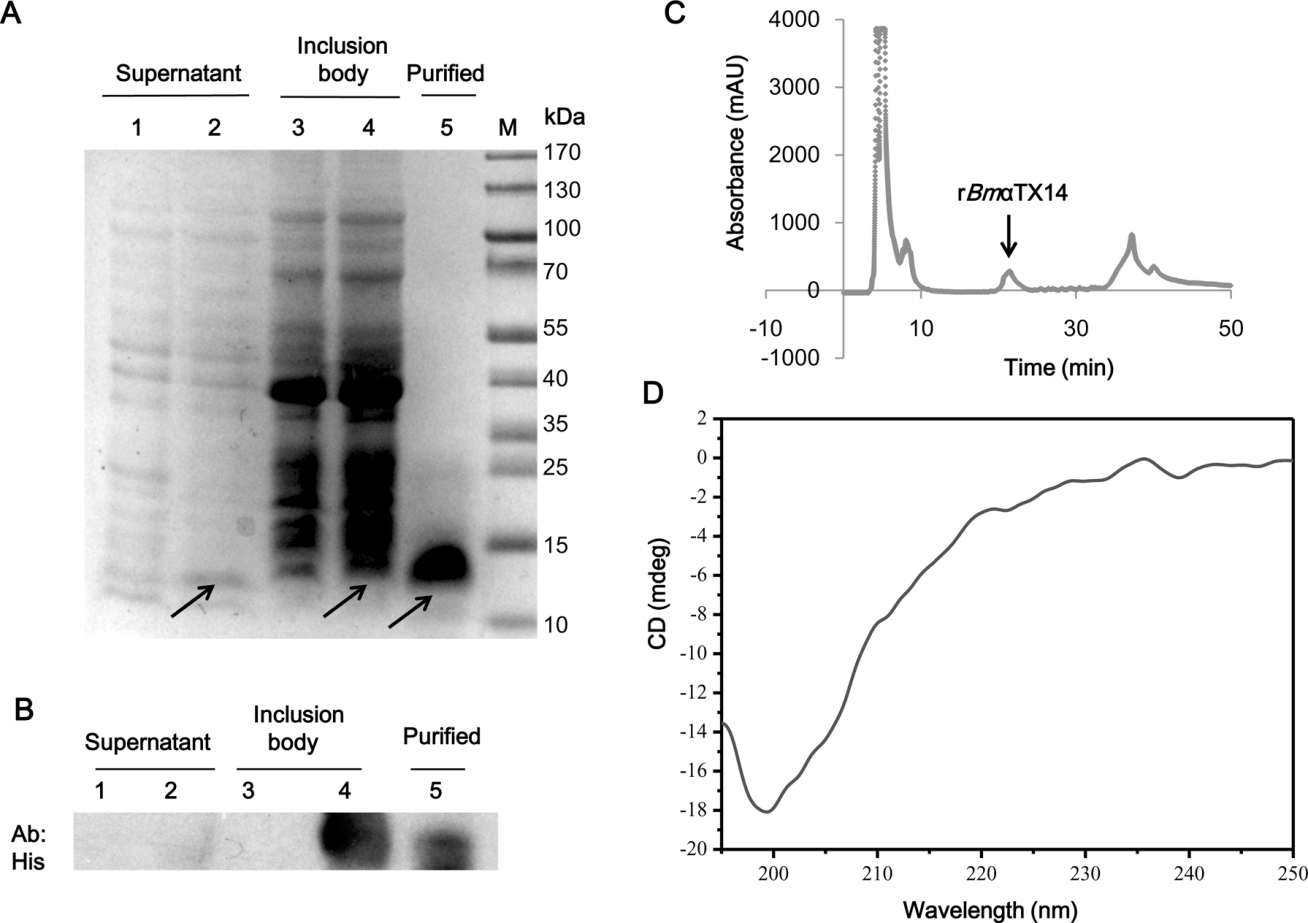
Expression analysis of r*Bmα*TX14 in *E*. *coli*. **(**A) Tricine-SDS-PAGE analysis of the expression and purification of 6-His-r*Bmα*TX14. Lanes 1 and 3 indicated cell lysate from *E*.*coli* with pET28a, lanes 2 and 4 indicated cell lysate from *E*.*coli* with pET28a-r*Bmα*TX14, and lane 5 is HPLC-purified r*Bmα*TX14. The arrows indicate the expressed protein. **(**B) Western blot analysis of r*Bmα*TX14 expression in *E*. *coli*. Lanes 1 and 3 indicated cell lysate from *E*.*coli* with pET28a, lanes 2 and 4 indicated cell lysate from *E*.*coli* with pET28a-r*Bmα*TX14, and lane 5 is HPLC-purified r*Bmα*TX14. The primary antibody utilized was anti-6-His. (C) Purification of r*Bmα*TX14 by RP-HPLC. The fraction containing r*Bmα*TX14, which peaked at 21 min, is indicated with the arrow. (D) The far-UV CD spectra of the 20 μM peptide were measured in the 195–250 nm wavelength range (protein secondary structure) on a Jasco J-810 spectropolarimeter. HPLC-purified r*Bmα*TX14 is comprised of approximately 43.8% of β-sheet, 12.9% β-turn, and 43.3% random coil.

To identify the r*Bmα*TX14 expression pattern in *E*.*coli*, we isolated protein samples from the cytoplasm and inclusion bodies. r*Bmα*TX14 was tagged with 6-His peptide; therefore, the samples were detected with the 6-His-tag antibody. As shown in Fig 1A & 1B, the antibody recognized an approximately 13 kDa protein, which is the predicted size of r*Bm*αTX14, in all isolated samples, showing that r*Bmα*TX14 exists both in the cytoplasm and inclusion bodies. Furthermore, HPLC was used to purify His-tagged r*Bm*αTX14 in this *E*. *coli* expression system, and a protein peak was observed (Fig 1C).

To determine the secondary peptide structure, we assessed the far-UV CD spectra of the peptide. As shown in Fig 1D, the far-UV CD spectrum of the peptide exhibited no obvious negative maxima at 222 and 208 nm, suggesting that an α-helical structure is not present. The secondary structure content analysis using Yang’s equation showed that the peptide is composed of approximately 43.8% β-sheet, 12.9% β-turn, and 43.3% random coil.

### r*Bmα*TX14 extended life span of *C*. *elegans*

**Fig 2:**
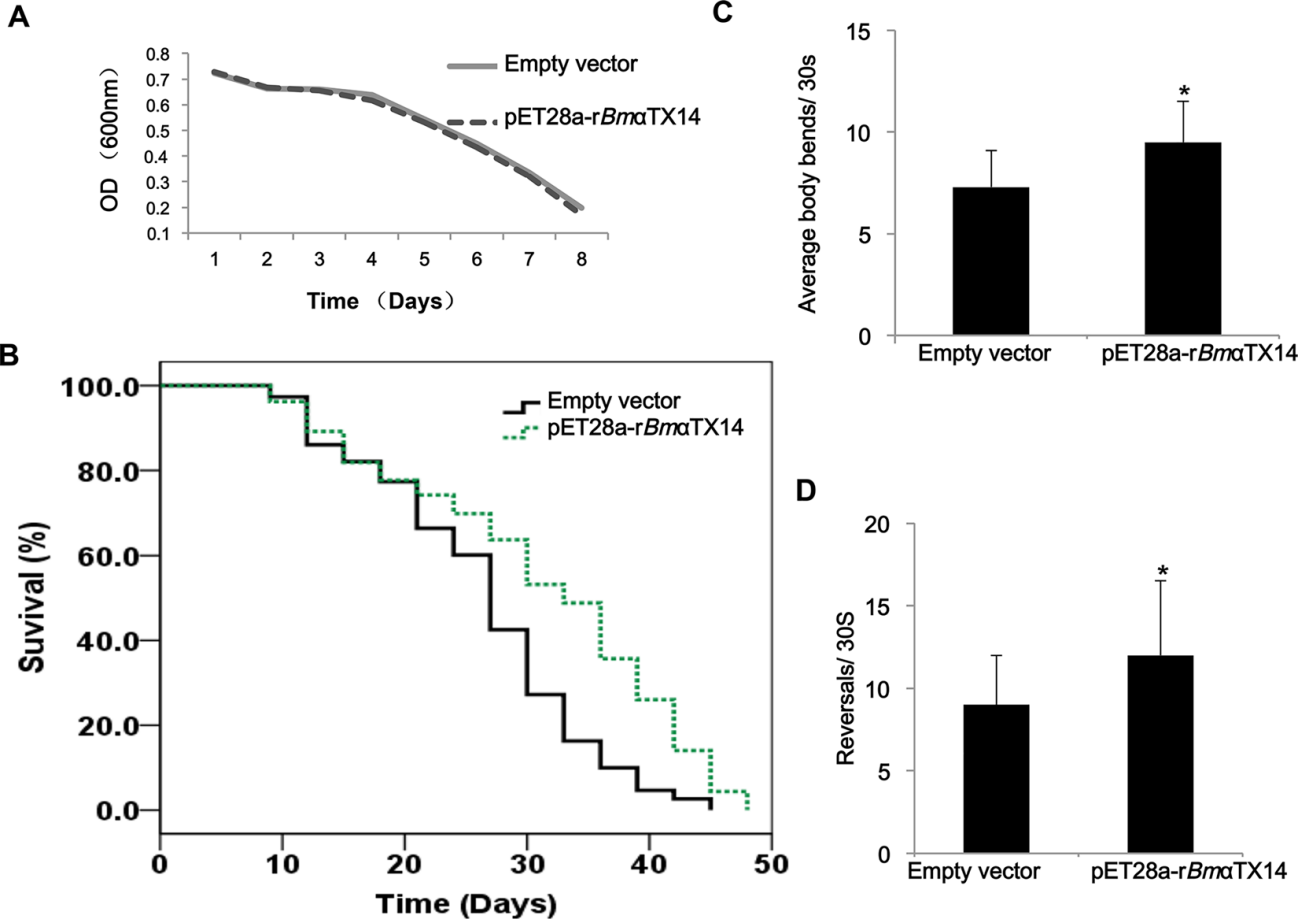
r*Bm*αTX14 extended the life span and promoted the locomotion of *C*. *elegans*. (A) Food clearance assay demonstrating the effects of pET28a-r*Bm*αTX14 on nematode growth and reproduction. The absorbance (600 nm) was measured daily for a week. (B) pET28a-r*Bm*αTX14 increased the *C*. *elegans* life span. The nematodes were treated with or without pET28a-r*Bm*αTX14 in 96-well plates. The survival data were plotted using the Kaplan-Meier method and analyzed by log-rank tests using SPSS 17.0 software. Body bend frequency (C) and reversals (D) were analyzed using a SAMSUNG SCC-101BP device and VideoMach software (Data were expressed as mean values ± SD, *p < 0.05). 100 worms were observed for each condition in motility assays, and the data represent an average of at least three independent experiments.

Expressed r*Bm*αTX14 in *E*. *coli* retained its biological structure; therefore, a food clearance assay was conducted to test whether r*Bm*αTX14 had any effects on *C*. *elegans* growth and reproduction. Taking advantage of the short life cycle and the ability of *C*. *elegans* to grow in *E*. *coli* liquid culture, the *E*. *coli* stains were used to feed *C*. *elegans*. The rate at which the *E*. *coli* suspension (food source) was consumed in 7 days was approximately the same in the r*Bm*αTX14 and control group (Fig 2A), indicating that r*Bm*αTX14 has little impact on the growth and reproduction of *C*. *elegans*.

Longevity is an important characteristic in this animal model [26, 27]; therefore, we further tested the life span of nematodes treated with r*Bmα*TX14 or the control. As shown in Fig 2B, the life span in the r*Bmα*TX14 group was longer than that of the control by approximately 19.2% (S1 Table).

### r*Bmα*TX14 promoted locomotion of *C*. *elegans*

Locomotion is also an important characteristic of *C*. *elegans*; therefore, we examined two basic body movements to observe any behavioral changes of nematodes fed with r*Bmα*TX14 [28–30]. Both the average body bends frequency and reversals significantly increased in the r*Bmα*TX14 group compared with the control (Fig 2C & 2D), suggesting that r*Bmα*TX14 promotes locomotion.

### Genes involved in life span extension and locomotion promoting phenotype

*C*. *elegans* gene expression was monitored to observe any corresponding effects caused by the ingestion of r*Bmα*TX14 and to identify the genes underlying the observed phenotypes [31–33]. Therefore, we examined the gene expression of adult *C*. *elegans* using microarray expression profiling, which identified 178 genes that were up-regulated and 12 genes that were down-regulated in the r*Bmα*TX14-fed animals compared with the control (S2 Table). Many of these deregulated genes are involved in life span regulation or other interesting processes (Table 1). Among these genes, *abu-1*, *abu-5*, *abu-7*, *abu-8*, *and abu-11* belong to ABU family of genes[34, 35], and *abu-11* overexpression is sufficient to increase *C*. *elegans* survival [36]. Specifically, *ptr-23* has been previously reported to increase *C*. *elegans* life span via the *daf-2* pathway. Additionally, the pathways affected by the *C26B9*.*3* and *T19B10*.*2* RNAi clones also shorten life span and are related to the *daf-2* pathway [37].

**Table 1:** List of differentially expressed genes and their physiological functions.

Gene ID	Description	Biological process or function	Reference
*abu-1*, *abu-5*, *abu-7*, *abu-8*, *abu-11*	endoplasmic reticulum stress-family genes	defense to pathogen infection; positively regulate life span	[35] [36]
*pqn-74*, *pqn-91*	endoplasmic reticulum stress-family genes	RNAi of these genes shorten life span	[34]
*ptr-23*	a predicted plasma membrane	RNAi of this gene shorten life span	[37]
*M03F4*.*6*	unknown	RNAi clone shortens daf-2 (e1370) life span	[37]
*tag-297*	unknown	RNAi clone shortens daf-2 (e1370) life span	[37]
*C26B9*.*3*	unknown	RNAi clones producing accelerated aging	[37]
*T19B10*.*2*	unknown	RNAi clones producing accelerated aging	[37]
*F10D11*.*6*	a putative lipopolysaccharide-binding protein homologue	knockdown of the gene result in developmental defects and very early death	[38]
*dao-4*	aging-related gene	Unknown	[39]
*cav-1*	cytoplasmic membrane-anchored scaffolding gene	promoting tumor progression, vesicular transport, transformation	[40]
*nnt-1*	nicotinamide nucleotide, transhydrogenase gene	defense of oxidative stress, regulating tumor growth	[41]
*grd-14*	unknown	nematode Hh-related (Hh-r) protein in Hedgehog (Hh) signaling pathway	[42]
*wrt-4*	extracellular region, plasma membrane	nematode Hh-related (Hh-r) protein in Hedgehog (Hh) signaling pathway	[42]
*ugt-6*	UDP-glycosyl transferase	Detoxification	[43]
*fmo-1*	flavin-containing monoxygenase	Detoxification	[21]
*col-97*	extracellular matrix protein	locomotion-promoting	[44]
*C06G1*.*1*	unknown	Locomotion	[45]
*C34E7*.*4*	unknown	locomotion; positive regulation of multicellular organism growth	[46]
*lpr-3*	extracellular region	locomotion; nematode larval development	[46]

**Fig 3:**
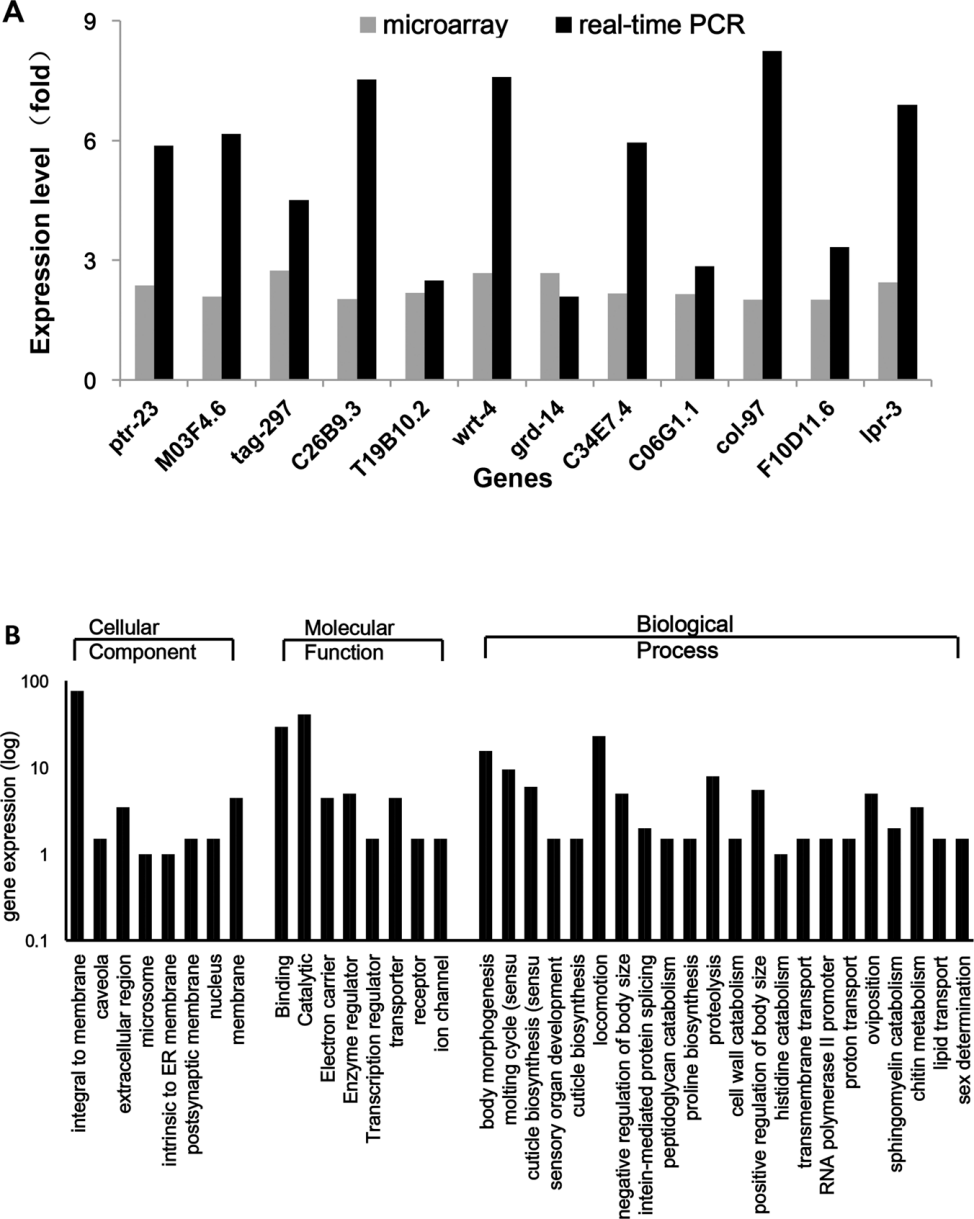
Relative expression of various genes in *C*. *elegans* fed with pET28a-r*Bm*αTX14. (A) The gene expression changes were detected through Affymetrix Genechip profiling. qRT-PCR analysis also confirms the differential regulation of genes identified through microarray analysis. qRT-PCR was used to examine changes in gene expression. (B) Molecular annotation system analysis of the genes. The differentially expressed genes were classificated of by GO-term annotation.

We further re-tested 16 of the up-regulated genes, which were chosen based on their roles in anti-aging and locomotion, using quantitative RT-PCR (qRT-PCR) analysis. The results showed that 12 out of the 16 tested genes showed similar changes using qRT-PCR and microarray analysis (Fig 3A). The primers utilized are listed in Table 2. Thus, our microarray analysis identified a small but reliable set of genes that are differentially expressed in animals fed with r*Bmα*TX14.

**Table 2:** The primers of selected genes for quantitative RT-PCR.

Gene symbols	Forward primers (5'-3')	Reverse primers (5'-3')
*ptr-23*	TCCTGTGCGGAGTTCGGTT	TTTGAGGGTTGACGGGTAA
*M03F4*.*6*	TTAGCACCCGCCAAAATC	ACAGAATCCCCGTTCAGTATC
*tag-297*	GCAGAAGCAAGGAGCAGTAGAT	CGAGACCCAGTATTCCAAGAGTT
*C26B9*.*3*	ACCGACCGAGTATTCGTTTC	AAGGGATGGATTGTTTGGAT
*T19B10*.*2*	GCGACTTGCTTTGCTTCC	TTGTGGCTTGCGTTCTCC
*wrt-4*	GGTTCAATACTGGCTTCATCG	CATCCTCAGAATAGGGCACA
*grd-14*	GCTTCTTCTTTTCGTCGCC	TGGTTTGTGAACTTGATGCTG
*C34E7*.*4*	GATTCTTTGCTCATCAAGTTCC	ATCGTTCTGGCTTTCCGT
*C06G1*.*1*	GACTTCCGCCTTCCATACC	CTTCAGATACCCAACCAACG
*col-97*	CTTCCGTCAGACTCCAAACA	TGGTCCAACTCCTCCATCA
*F10D11*.*6*	CTGTTCGTGCTCCATCTGTC	TCCAACTTTCGTGTTTGTCTG
*lpr-3*	ACTTACCCGCAATGACAAAA	CCACGGAAAGCATACCCA
*act-1*	CTCCTCACTGAAGCCCCACT	CTTGATGTCACGGACGATTT

The sequences of these genes were obtained from website of NCBI (National Center of Biotechnology Information, http://www.ncbi.nlm.nih.gov/) and the primers were designed by Primer 5 software.

Moreover, all 190 differentially expressed genes were analyzed using a free Molecular Annotation System 2.0 (MAS 2.0, www.capitalbio.com). With the MAS 2.0 tool, the pathways are ranked by statistical significance by calculating their p*-*values based on the hypergeometric distribution [32]. The classification of the differentially expressed genes by GO-term annotation also highlighted the genes involved in locomotion and other biological processes (Fig 3B).

### The H_15_ to F_15_ amino acid change in r*Bmα*TX14 alters the life-span extension of *C*. *elegans*

**Fig 4:**
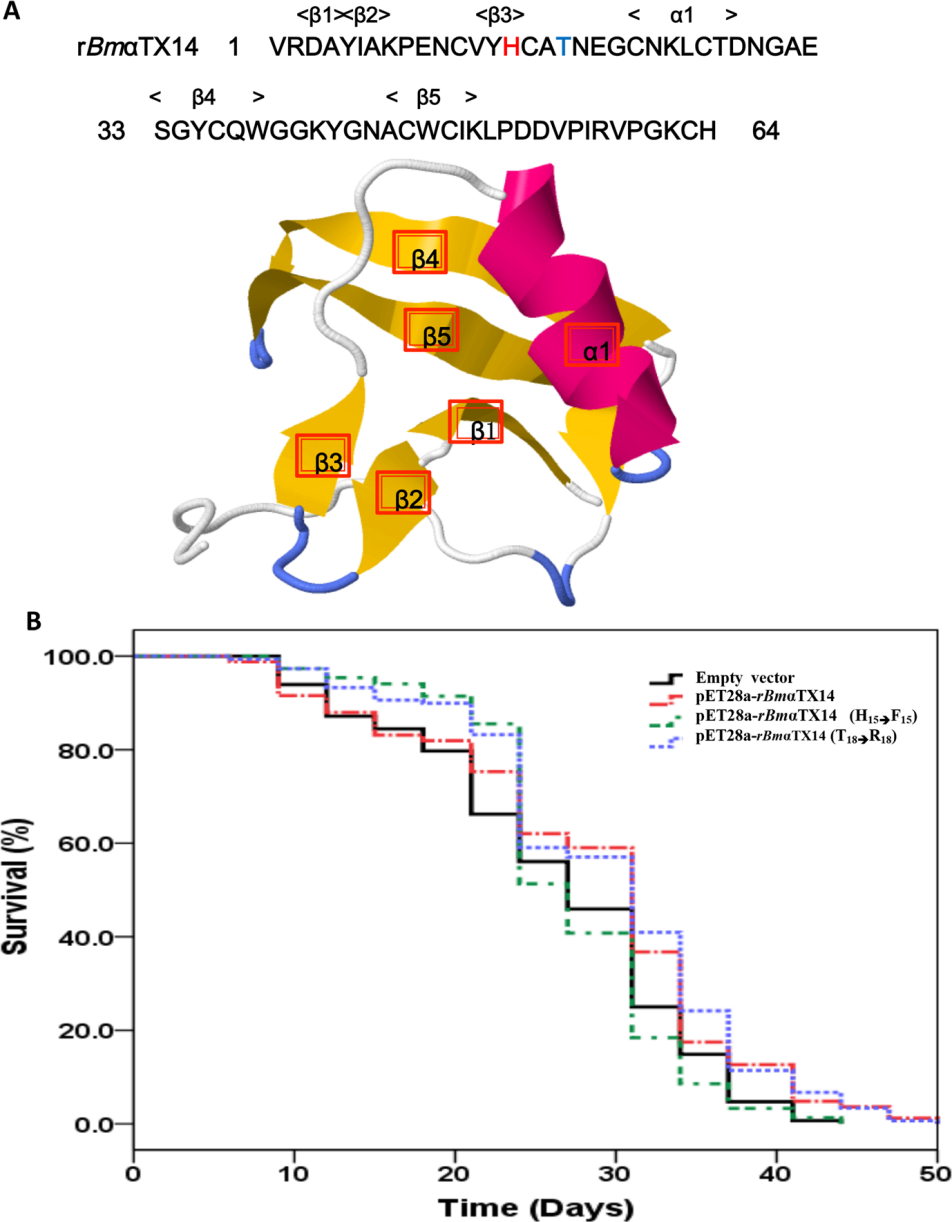
r*Bm*αTX14 mutations affect the extension of *C*. *elegans* life span. (A) The *rBm*αTX14 protein sequences were mutant at H_15_ or T_18_. The structure model predicted that H_15_ play an important role in β3. (B) Amino acid change from H_15_ to F_15_ in r*Bm*αTX14 altered the life span of *C*. *elegans*. The life span assays were performed as above, and the nematodes were treated with different mutant strains in 96-well plates. pET28a-r*Bmα*TX14 and pET28a-r*Bmα*TX14 (T_18⟶_R_18_) significantly increased the life span by 14.8% compared with empty vector and pET28a-r*Bmα*TX14 (H_15⟶_F_15_).

According to the analysis of the r*Bmα*TX14 structure, amino acid site mutations were created to identify r*Bmα*TX14 function. In the r*Bmα*TX14 sequence shown in Fig 4A, H_15_ was changed to F_15,_ and T_18_ was changed to R_18._ Especially the software predicted that H_15_ was involved in the β-sheet. After the mutant proteins were expressed in *E*. *coli* strains, these bacteria were fed to *C*. *elegans*.

Given the impact that r*Bmα*TX14 has on *C*. *elegans* survival, we wondered whether key r*Bmα*TX14 amino acids influence longevity. We feed the animals with appropriate strains, and found that pET28a-r*Bmα*TX14 and pET28a-r*Bmα*TX14 (T_18_⟶R_18_) increased the median day of life span by 14.8% compared with empty vector and pET28a-r*Bmα*TX14 (H_15_⟶F_15_). P-value indicated this change was statistically significant. Specifically, the life span with pET28a-r*Bmα*TX14 and pET28a-r*Bmα*TX14 (T_18_⟶R_18_) were approximately 50 days in length, while the life spans with empty vector and pET28a-r*Bmα*TX14 (H_15_⟶F_15_) were approximately 44 days in length (Fig 4B, Table 3 and S3 Table). Then H_15_ was shown to be an important amino acid site because when the amino site was mutated to F_15_, the effect of r*Bmα*TX14 on the life span was lost. However, if the mutation site was T_18_, no significant effects on r*Bmα*TX14 function were observed.

**Table 3:** Effect of r*Bmα*TX14 mutations on longevity.

Strain	Treatment	Mean life span±SEM (days)	Median life span±SEM (days)	Maximum life spans (days)	*p*-value vs. control	Life span extension	Number of animal
N2	Empty vector	26±0.7	27±0.9	44	-	-	148
N2	pET28a-r*Bmα*TX14	28±0.8	31±0.7	50	0.008	14.8%	166
N2	pET28a-r*Bm*αTX14 (H15⟶F15)	27±0.5	27±0.5	44	0.717	-	152
N2	pET28a-r*Bmα*TX14 (T18⟶R18)	29±0.7	31±1	50	0.001	14.8%	149

Life span analysis of empty vector, pET28a-r*Bmα*TX14, pET28a-r*Bmα*TX14 (T_18⟶_R_18_), pET28a-r*Bmα*TX14 (H_15⟶_F_15_) was starting from L4. These combined results were derived from individual experiments that are described in S3 Table. P-values indicate comparisons between expression plasmids and empty vector. N2 represents the wild type *C*. *elegans*. SEM = standard error of the mean. *p*-values (log-rank test) refer to the control experiment.

## Discussion

Despite the extensive study of the effects of scorpion venom toxins on ion channels, why scorpion can also be eaten as food or medicine remains unknown. Scorpion bioactivities such as anti-aging and anti-tumor properties remain to be uncovered. To test the effects of r*Bmα*TX14 as food, *C*. *elegans* was used here as the animal model to screen the peptides. *C*. *elegans* has a short life-cycle, small size and ease of culturing and is extensively used as an animal model, especially for the detailed analysis of the molecular pathways involved in aging and other physiological activities [18].

Although r*Bmα*TX14 showed no obvious effects on growth and reproduction, r*Bmα*TX14 extended *C*. *elegans* life span and promoted locomotion. To understand the underlying mechanisms, microarray analysis was widely used to screen the genes involved in the pathways [47]. Here, the potential genes that regulate this mechanism are listed in Table 2, including *ptr-23*, *M03F4*.*6*, *tag-297*, *C26B9*.*3* and others. In addition to these genes, the *M03F4*.*6* and *tag-297* RNAi clones shortened life span and produced other pleiotropic effects that may shorten life span [37]. Furthermore, r*Bmα*TX14 promoted *C*. *elegans* locomotion, and genes such as *wrt-4*, *grd-14*, *C06G1*.*1*, *col-97*, *F10D11*.*6* and *lpr-3* are known to be involved in nematode locomotion.

Interestingly, r*Bmα*TX14 induced the expression of *cav-1* and *nnt-1*, which play various roles in tumor progression. Cav-1 protein levels are consistently down-regulated in a wide range of human cancers, including ovarian carcinomas, sarcomas, and mammary carcinomas [48]. These genes also have *in vivo* tumor suppressor properties in certain tissues such as the mammary gland and murine animal models [40, 49]. *nnt-1* also encoded a nicotinamide nucleotide transhydrogenase, which normally functions to maintain electron transport chain activity. Reducing the activity of this gene caused a metabolic shift that promotes tumor growth [41], and wide variety genes extend *C*. *elegans* life span and also reduce tumor cell division [50], implying that *rBmα*TX14 may have a valuable use in cancer therapy.

Since starvation could elongate the life span of nematodes [51], calorically/nutritionally restricted might affect the nematodes. However, the data showed the life span of *C*. *elegans* feeding with pET28a-r*Bmα*TX14 was longer than with pET28a-r*Bmα*TX14 (NEG) (S4 Table and S1 Fig). Because pET28a-r*Bmα*TX14 and pET28a-r*Bmα*TX14 (NEG) all have inclusion bodies, so calorically/nutritionally restricted or biomass should not be the main reason that affects gene transcription. Maybe the peptides were digested by proteases in the gut and absorbed by the gut of the animal. Though *Bmα*TX14 is known to be a potent blocker of the sodium channel, genome sequence analysis showed that voltage-gated sodium channel was absent in *C*. *elegans*. In the muscle cells of *C*. *elegans*, voltage-gated calcium channels had the similar functions as well as the sodium channel [52–54]. Then it is still interesting to know if the potential working mechanism of r*Bmα*TX14 is through the related voltage-gated channel?

Overall, in this study, we’ve expressed, purified and measured the secondary structure of recombinant protein r*Bmα*TX14. Feeding the nematodes with pET28a-r*Bmα*TX14, we’ve demonstrated that r*Bmα*TX14 caused extension of the life span and promoted the locomotion of the nematodes. Further investigation uncovered the specific genes that play various roles in the life span and locomotion of *C*. *elegans*. In addition, the amino acid site H_15_ of r*Bmα*TX14 was proved to be an important site in the function of the protein. This interesting finding may provide an insight into the utility of scorpion venom in anti-aging as food or medicine.

## Supporting Information

S1 Fig pET28a-r*Bmα*TX14 (NEG) had no effects on the life-span extension and the locomotion promotion of *C*. *elegans*. (A) pET28a-r*Bm*αTX14 (NEG) did not increase the *C*. *elegans* life span. The nematodes were treated with empty vector, pET28a-r*Bm*αTX14 and pET28a-r*Bm*αTX14 (NEG) in 96-well plates. The survival data were plotted using the Kaplan-Meier method and analyzed by log-rank tests using SPSS 17.0 software. Body bend frequency (B) and reversals (C) were analyzed using a SAMSUNG SCC-101BP device and VideoMach software (Data were expressed as mean values ± SD, *p < 0.05). 100 worms were observed for each condition in motility assays, and the data represent an average of at least three independent experiments. (D) Expression of r*Bmα*TX14 and the negative control in *E*. *coli*. Lanes 1 and 3 indicated cell lysate from *E*.*coli* with pET28a, lanes 2 and 4 indicated cell lysate from *E*.*coli* with pET28a-r*Bmα*TX14, and lane 5 and 6 indicated cell lysate from *E*.*coli* with pET28a-r*Bm*αTX14 (NEG). The primary antibody utilized was anti-6-His.
(TIF)

S1 Table pET28a-r*Bmα*TX14 extended the life span of *C*. *elegans*. Group 1 is *C*. *elegans* fed with empty vector, and Group 2 is *C*. *elegans* fed with pET28a-r*Bmα*TX14.
(DOC)

S2 Table The different genes in *C*. *elegans* fed with pET28a-r*Bmα*TX14 through Affymetrix Microarray analysis. Gene lists were for 171 Up and 11 Down regulation genes.
(DOC)

S3 Table Amino acid mutations of r*Bmα*TX14 affect the life span extension of *C*. *elegans*. Group 1 is *C*. *elegans* fed with control *E*.*coli* strain with empty vector, Group 2 is *C*. *elegans* fed with pET28a-r*Bmα*TX14, Group 3 is *C*. *elegans* fed with pET28a-r*Bmα*TX14 (H_15_⟶F_15_), and Group 4 is *C*. *elegans* fed with pET28a-r*Bmα*TX14 (T_18⟶_R_18_).
(DOC)

S4 Table Effects of pET28a-r*Bmα*TX14 and pET28a-r*Bmα*TX14 (NEG) on longevity. Life span analysis of empty vector, pET28a-r*Bmα*TX14 and pET28a-r*Bmα*TX14 (NEG) were starting from L4.
(DOC)

## Abbreviations

*C. elegans*: *Caenorhabditis elegans*
IPTG: isopropyl-β-D-thiogalactopyranoside
SDS: sodium dodecyl sulfate
PAGE: polyacrylamide gelelectrophoresis
CD: Circular dichroism spectroscopy
RT-PCR: reverse transcription-polymerase chain reaction
DMSO: dimethyl sulfoxide
MAS: Molecule Annotation System
