# Supplementary figures and images for: rBmαTX14 Increases the Life Span and Promotes the Locomotion of Caenorhabditis Elegans
Source: PLoS One. 2016 Sep 9;11(9):e0161847. doi: 10.1371/journal.pone.0161847 (PMC5017660; doi:10.1371/journal.pone.0161847)

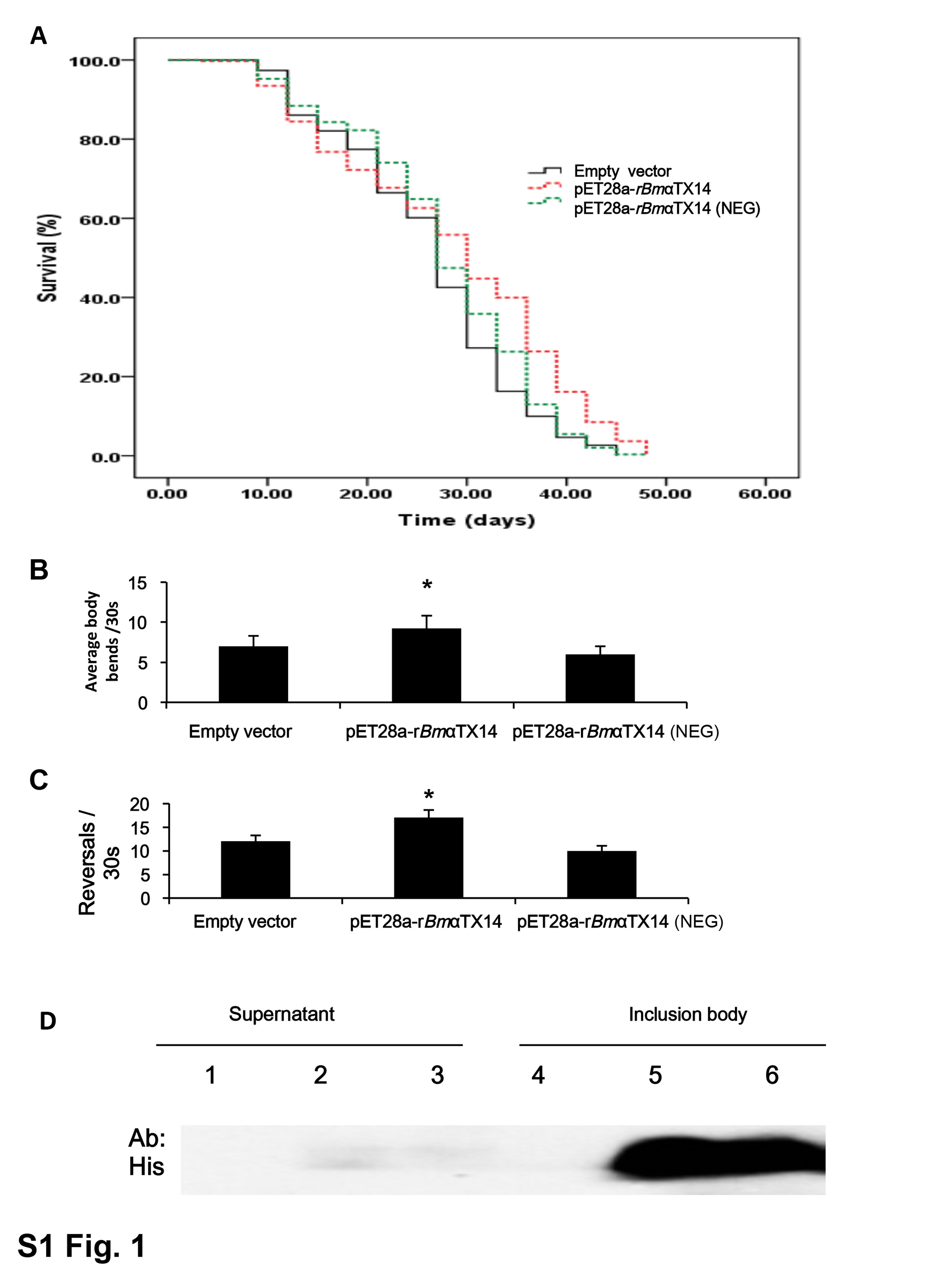

Supplement: S1 Fig — (A) pET28a-rBmαTX14 (NEG) did not increase the C. elegans life span. The nematodes were treated with empty vector, pET28a-rBmαTX14 and pET28a-rBmαTX14 (NEG) in 96-well plates. The survival data were plotted using the Kaplan-Meier method and analyzed by log-rank tests using SPSS 17.0 software. Body bend frequency (B) and reversals (C) were analyzed using a SAMSUNG SCC-101BP device and VideoMach software (Data were expressed as mean values ± SD, *p < 0.05). 100 worms were observed for each condition in motility assays, and the data represent an average of at least three independent experiments. (D) Expression of rBmαTX14 and the negative control in E. coli. Lanes 1 and 3 indicated cell lysate from E.coli with pET28a, lanes 2 and 4 indicated cell lysate from E.coli with pET28a-rBmαTX14, and lane 5 and 6 indicated cell lysate from E.coli with pET28a-rBmαTX14 (NEG). The primary antibody utilized was anti-6-His. (TIF) [file pone.0161847.s001.tif]
