# Supplementary material for: rBmαTX14 Increases the Life Span and Promotes the Locomotion of Caenorhabditis Elegans
Source: PLoS One. 2016 Sep 9;11(9):e0161847. doi: 10.1371/journal.pone.0161847 (PMC5017660; doi:10.1371/journal.pone.0161847)
Supplement: S1 Table — Group 1 is C. elegans fed with empty vector, and Group 2 is C. elegans fed with pET28a-rBmαTX14. (DOC) [file pone.0161847.s002.doc]

**S1 Table. r*Bm*αTX14 extended the life span of *C. elegans.***

**Kaplan-Meier**

| **Case Processing Summary** | | | | |
| --- | --- | --- | --- | --- |
| group |  | | Censored | |
|  | Total N | N of Events | N | Percent |
| 1.00 | 301 | 301 | 0 | .0% |
| 2.00 | 342 | 342 | 0 | .0% |
| Overall | 643 | 643 | 0 | .0% |

| **Survival Table** | | | | | | | |
| --- | --- | --- | --- | --- | --- | --- | --- |
| g | |  | | Cumulative Proportion Surviving at the Time | |  | |
|  |  | Time | Status | Estimate | Std. Error | N of Cumulative Events | N of Remaining Cases |
| 1.00 | 1 | 9.000 | 1.00 | . | . | 1 | 300 |
|  | 2 | 9.000 | 1.00 | . | . | 2 | 299 |
|  | 3 | 9.000 | 1.00 | . | . | 3 | 298 |
|  | 4 | 9.000 | 1.00 | . | . | 4 | 297 |
|  | 5 | 9.000 | 1.00 | . | . | 5 | 296 |
|  | 6 | 9.000 | 1.00 | . | . | 6 | 295 |
|  | 7 | 9.000 | 1.00 | . | . | 7 | 294 |
|  | 8 | 9.000 | 1.00 | .973 | .009 | 8 | 293 |
|  | 9 | 12.000 | 1.00 | . | . | 9 | 292 |
|  | 10 | 12.000 | 1.00 | . | . | 10 | 291 |
|  | 11 | 12.000 | 1.00 | . | . | 11 | 290 |
|  | 12 | 12.000 | 1.00 | . | . | 12 | 289 |
|  | 13 | 12.000 | 1.00 | . | . | 13 | 288 |
|  | 14 | 12.000 | 1.00 | . | . | 14 | 287 |
|  | 15 | 12.000 | 1.00 | . | . | 15 | 286 |
|  | 16 | 12.000 | 1.00 | . | . | 16 | 285 |
|  | 17 | 12.000 | 1.00 | . | . | 17 | 284 |
|  | 18 | 12.000 | 1.00 | . | . | 18 | 283 |
|  | 19 | 12.000 | 1.00 | . | . | 19 | 282 |
|  | 20 | 12.000 | 1.00 | . | . | 20 | 281 |
|  | 21 | 12.000 | 1.00 | . | . | 21 | 280 |
|  | 22 | 12.000 | 1.00 | . | . | 22 | 279 |
|  | 23 | 12.000 | 1.00 | . | . | 23 | 278 |
|  | 24 | 12.000 | 1.00 | . | . | 24 | 277 |
|  | 25 | 12.000 | 1.00 | . | . | 25 | 276 |
|  | 26 | 12.000 | 1.00 | . | . | 26 | 275 |
|  | 27 | 12.000 | 1.00 | . | . | 27 | 274 |
|  | 28 | 12.000 | 1.00 | . | . | 28 | 273 |
|  | 29 | 12.000 | 1.00 | . | . | 29 | 272 |
|  | 30 | 12.000 | 1.00 | . | . | 30 | 271 |
|  | 31 | 12.000 | 1.00 | . | . | 31 | 270 |
|  | 32 | 12.000 | 1.00 | . | . | 32 | 269 |
|  | 33 | 12.000 | 1.00 | . | . | 33 | 268 |
|  | 34 | 12.000 | 1.00 | . | . | 34 | 267 |
|  | 35 | 12.000 | 1.00 | . | . | 35 | 266 |
|  | 36 | 12.000 | 1.00 | . | . | 36 | 265 |
|  | 37 | 12.000 | 1.00 | . | . | 37 | 264 |
|  | 38 | 12.000 | 1.00 | . | . | 38 | 263 |
|  | 39 | 12.000 | 1.00 | . | . | 39 | 262 |
|  | 40 | 12.000 | 1.00 | . | . | 40 | 261 |
|  | 41 | 12.000 | 1.00 | . | . | 41 | 260 |
|  | 42 | 12.000 | 1.00 | .860 | .020 | 42 | 259 |
|  | 43 | 15.000 | 1.00 | . | . | 43 | 258 |
|  | 44 | 15.000 | 1.00 | . | . | 44 | 257 |
|  | 45 | 15.000 | 1.00 | . | . | 45 | 256 |
|  | 46 | 15.000 | 1.00 | . | . | 46 | 255 |
|  | 47 | 15.000 | 1.00 | . | . | 47 | 254 |
|  | 48 | 15.000 | 1.00 | . | . | 48 | 253 |
|  | 49 | 15.000 | 1.00 | . | . | 49 | 252 |
|  | 50 | 15.000 | 1.00 | . | . | 50 | 251 |
|  | 51 | 15.000 | 1.00 | . | . | 51 | 250 |
|  | 52 | 15.000 | 1.00 | . | . | 52 | 249 |
|  | 53 | 15.000 | 1.00 | . | . | 53 | 248 |
|  | 54 | 15.000 | 1.00 | .821 | .022 | 54 | 247 |
|  | 55 | 18.000 | 1.00 | . | . | 55 | 246 |
|  | 56 | 18.000 | 1.00 | . | . | 56 | 245 |
|  | 57 | 18.000 | 1.00 | . | . | 57 | 244 |
|  | 58 | 18.000 | 1.00 | . | . | 58 | 243 |
|  | 59 | 18.000 | 1.00 | . | . | 59 | 242 |
|  | 60 | 18.000 | 1.00 | . | . | 60 | 241 |
|  | 61 | 18.000 | 1.00 | . | . | 61 | 240 |
|  | 62 | 18.000 | 1.00 | . | . | 62 | 239 |
|  | 63 | 18.000 | 1.00 | . | . | 63 | 238 |
|  | 64 | 18.000 | 1.00 | . | . | 64 | 237 |
|  | 65 | 18.000 | 1.00 | . | . | 65 | 236 |
|  | 66 | 18.000 | 1.00 | . | . | 66 | 235 |
|  | 67 | 18.000 | 1.00 | . | . | 67 | 234 |
|  | 68 | 18.000 | 1.00 | .774 | .024 | 68 | 233 |
|  | 69 | 21.000 | 1.00 | . | . | 69 | 232 |
|  | 70 | 21.000 | 1.00 | . | . | 70 | 231 |
|  | 71 | 21.000 | 1.00 | . | . | 71 | 230 |
|  | 72 | 21.000 | 1.00 | . | . | 72 | 229 |
|  | 73 | 21.000 | 1.00 | . | . | 73 | 228 |
|  | 74 | 21.000 | 1.00 | . | . | 74 | 227 |
|  | 75 | 21.000 | 1.00 | . | . | 75 | 226 |
|  | 76 | 21.000 | 1.00 | . | . | 76 | 225 |
|  | 77 | 21.000 | 1.00 | . | . | 77 | 224 |
|  | 78 | 21.000 | 1.00 | . | . | 78 | 223 |
|  | 79 | 21.000 | 1.00 | . | . | 79 | 222 |
|  | 80 | 21.000 | 1.00 | . | . | 80 | 221 |
|  | 81 | 21.000 | 1.00 | . | . | 81 | 220 |
|  | 82 | 21.000 | 1.00 | . | . | 82 | 219 |
|  | 83 | 21.000 | 1.00 | . | . | 83 | 218 |
|  | 84 | 21.000 | 1.00 | . | . | 84 | 217 |
|  | 85 | 21.000 | 1.00 | . | . | 85 | 216 |
|  | 86 | 21.000 | 1.00 | . | . | 86 | 215 |
|  | 87 | 21.000 | 1.00 | . | . | 87 | 214 |
|  | 88 | 21.000 | 1.00 | . | . | 88 | 213 |
|  | 89 | 21.000 | 1.00 | . | . | 89 | 212 |
|  | 90 | 21.000 | 1.00 | . | . | 90 | 211 |
|  | 91 | 21.000 | 1.00 | . | . | 91 | 210 |
|  | 92 | 21.000 | 1.00 | . | . | 92 | 209 |
|  | 93 | 21.000 | 1.00 | . | . | 93 | 208 |
|  | 94 | 21.000 | 1.00 | . | . | 94 | 207 |
|  | 95 | 21.000 | 1.00 | . | . | 95 | 206 |
|  | 96 | 21.000 | 1.00 | . | . | 96 | 205 |
|  | 97 | 21.000 | 1.00 | . | . | 97 | 204 |
|  | 98 | 21.000 | 1.00 | . | . | 98 | 203 |
|  | 99 | 21.000 | 1.00 | . | . | 99 | 202 |
|  | 100 | 21.000 | 1.00 | . | . | 100 | 201 |
|  | 101 | 21.000 | 1.00 | .664 | .027 | 101 | 200 |
|  | 102 | 24.000 | 1.00 | . | . | 102 | 199 |
|  | 103 | 24.000 | 1.00 | . | . | 103 | 198 |
|  | 104 | 24.000 | 1.00 | . | . | 104 | 197 |
|  | 105 | 24.000 | 1.00 | . | . | 105 | 196 |
|  | 106 | 24.000 | 1.00 | . | . | 106 | 195 |
|  | 107 | 24.000 | 1.00 | . | . | 107 | 194 |
|  | 108 | 24.000 | 1.00 | . | . | 108 | 193 |
|  | 109 | 24.000 | 1.00 | . | . | 109 | 192 |
|  | 110 | 24.000 | 1.00 | . | . | 110 | 191 |
|  | 111 | 24.000 | 1.00 | . | . | 111 | 190 |
|  | 112 | 24.000 | 1.00 | . | . | 112 | 189 |
|  | 113 | 24.000 | 1.00 | . | . | 113 | 188 |
|  | 114 | 24.000 | 1.00 | . | . | 114 | 187 |
|  | 115 | 24.000 | 1.00 | . | . | 115 | 186 |
|  | 116 | 24.000 | 1.00 | . | . | 116 | 185 |
|  | 117 | 24.000 | 1.00 | . | . | 117 | 184 |
|  | 118 | 24.000 | 1.00 | . | . | 118 | 183 |
|  | 119 | 24.000 | 1.00 | . | . | 119 | 182 |
|  | 120 | 24.000 | 1.00 | .601 | .028 | 120 | 181 |
|  | 121 | 27.000 | 1.00 | . | . | 121 | 180 |
|  | 122 | 27.000 | 1.00 | . | . | 122 | 179 |
|  | 123 | 27.000 | 1.00 | . | . | 123 | 178 |
|  | 124 | 27.000 | 1.00 | . | . | 124 | 177 |
|  | 125 | 27.000 | 1.00 | . | . | 125 | 176 |
|  | 126 | 27.000 | 1.00 | . | . | 126 | 175 |
|  | 127 | 27.000 | 1.00 | . | . | 127 | 174 |
|  | 128 | 27.000 | 1.00 | . | . | 128 | 173 |
|  | 129 | 27.000 | 1.00 | . | . | 129 | 172 |
|  | 130 | 27.000 | 1.00 | . | . | 130 | 171 |
|  | 131 | 27.000 | 1.00 | . | . | 131 | 170 |
|  | 132 | 27.000 | 1.00 | . | . | 132 | 169 |
|  | 133 | 27.000 | 1.00 | . | . | 133 | 168 |
|  | 134 | 27.000 | 1.00 | . | . | 134 | 167 |
|  | 135 | 27.000 | 1.00 | . | . | 135 | 166 |
|  | 136 | 27.000 | 1.00 | . | . | 136 | 165 |
|  | 137 | 27.000 | 1.00 | . | . | 137 | 164 |
|  | 138 | 27.000 | 1.00 | . | . | 138 | 163 |
|  | 139 | 27.000 | 1.00 | . | . | 139 | 162 |
|  | 140 | 27.000 | 1.00 | . | . | 140 | 161 |
|  | 141 | 27.000 | 1.00 | . | . | 141 | 160 |
|  | 142 | 27.000 | 1.00 | . | . | 142 | 159 |
|  | 143 | 27.000 | 1.00 | . | . | 143 | 158 |
|  | 144 | 27.000 | 1.00 | . | . | 144 | 157 |
|  | 145 | 27.000 | 1.00 | . | . | 145 | 156 |
|  | 146 | 27.000 | 1.00 | . | . | 146 | 155 |
|  | 147 | 27.000 | 1.00 | . | . | 147 | 154 |
|  | 148 | 27.000 | 1.00 | . | . | 148 | 153 |
|  | 149 | 27.000 | 1.00 | . | . | 149 | 152 |
|  | 150 | 27.000 | 1.00 | . | . | 150 | 151 |
|  | 151 | 27.000 | 1.00 | . | . | 151 | 150 |
|  | 152 | 27.000 | 1.00 | . | . | 152 | 149 |
|  | 153 | 27.000 | 1.00 | . | . | 153 | 148 |
|  | 154 | 27.000 | 1.00 | . | . | 154 | 147 |
|  | 155 | 27.000 | 1.00 | . | . | 155 | 146 |
|  | 156 | 27.000 | 1.00 | . | . | 156 | 145 |
|  | 157 | 27.000 | 1.00 | . | . | 157 | 144 |
|  | 158 | 27.000 | 1.00 | . | . | 158 | 143 |
|  | 159 | 27.000 | 1.00 | . | . | 159 | 142 |
|  | 160 | 27.000 | 1.00 | . | . | 160 | 141 |
|  | 161 | 27.000 | 1.00 | . | . | 161 | 140 |
|  | 162 | 27.000 | 1.00 | . | . | 162 | 139 |
|  | 163 | 27.000 | 1.00 | . | . | 163 | 138 |
|  | 164 | 27.000 | 1.00 | . | . | 164 | 137 |
|  | 165 | 27.000 | 1.00 | . | . | 165 | 136 |
|  | 166 | 27.000 | 1.00 | . | . | 166 | 135 |
|  | 167 | 27.000 | 1.00 | . | . | 167 | 134 |
|  | 168 | 27.000 | 1.00 | . | . | 168 | 133 |
|  | 169 | 27.000 | 1.00 | . | . | 169 | 132 |
|  | 170 | 27.000 | 1.00 | . | . | 170 | 131 |
|  | 171 | 27.000 | 1.00 | . | . | 171 | 130 |
|  | 172 | 27.000 | 1.00 | . | . | 172 | 129 |
|  | 173 | 27.000 | 1.00 | .425 | .028 | 173 | 128 |
|  | 174 | 30.000 | 1.00 | . | . | 174 | 127 |
|  | 175 | 30.000 | 1.00 | . | . | 175 | 126 |
|  | 176 | 30.000 | 1.00 | . | . | 176 | 125 |
|  | 177 | 30.000 | 1.00 | . | . | 177 | 124 |
|  | 178 | 30.000 | 1.00 | . | . | 178 | 123 |
|  | 179 | 30.000 | 1.00 | . | . | 179 | 122 |
|  | 180 | 30.000 | 1.00 | . | . | 180 | 121 |
|  | 181 | 30.000 | 1.00 | . | . | 181 | 120 |
|  | 182 | 30.000 | 1.00 | . | . | 182 | 119 |
|  | 183 | 30.000 | 1.00 | . | . | 183 | 118 |
|  | 184 | 30.000 | 1.00 | . | . | 184 | 117 |
|  | 185 | 30.000 | 1.00 | . | . | 185 | 116 |
|  | 186 | 30.000 | 1.00 | . | . | 186 | 115 |
|  | 187 | 30.000 | 1.00 | . | . | 187 | 114 |
|  | 188 | 30.000 | 1.00 | . | . | 188 | 113 |
|  | 189 | 30.000 | 1.00 | . | . | 189 | 112 |
|  | 190 | 30.000 | 1.00 | . | . | 190 | 111 |
|  | 191 | 30.000 | 1.00 | . | . | 191 | 110 |
|  | 192 | 30.000 | 1.00 | . | . | 192 | 109 |
|  | 193 | 30.000 | 1.00 | . | . | 193 | 108 |
|  | 194 | 30.000 | 1.00 | . | . | 194 | 107 |
|  | 195 | 30.000 | 1.00 | . | . | 195 | 106 |
|  | 196 | 30.000 | 1.00 | . | . | 196 | 105 |
|  | 197 | 30.000 | 1.00 | . | . | 197 | 104 |
|  | 198 | 30.000 | 1.00 | . | . | 198 | 103 |
|  | 199 | 30.000 | 1.00 | . | . | 199 | 102 |
|  | 200 | 30.000 | 1.00 | . | . | 200 | 101 |
|  | 201 | 30.000 | 1.00 | . | . | 201 | 100 |
|  | 202 | 30.000 | 1.00 | . | . | 202 | 99 |
|  | 203 | 30.000 | 1.00 | . | . | 203 | 98 |
|  | 204 | 30.000 | 1.00 | . | . | 204 | 97 |
|  | 205 | 30.000 | 1.00 | . | . | 205 | 96 |
|  | 206 | 30.000 | 1.00 | . | . | 206 | 95 |
|  | 207 | 30.000 | 1.00 | . | . | 207 | 94 |
|  | 208 | 30.000 | 1.00 | . | . | 208 | 93 |
|  | 209 | 30.000 | 1.00 | . | . | 209 | 92 |
|  | 210 | 30.000 | 1.00 | . | . | 210 | 91 |
|  | 211 | 30.000 | 1.00 | . | . | 211 | 90 |
|  | 212 | 30.000 | 1.00 | . | . | 212 | 89 |
|  | 213 | 30.000 | 1.00 | . | . | 213 | 88 |
|  | 214 | 30.000 | 1.00 | . | . | 214 | 87 |
|  | 215 | 30.000 | 1.00 | . | . | 215 | 86 |
|  | 216 | 30.000 | 1.00 | . | . | 216 | 85 |
|  | 217 | 30.000 | 1.00 | . | . | 217 | 84 |
|  | 218 | 30.000 | 1.00 | . | . | 218 | 83 |
|  | 219 | 30.000 | 1.00 | .272 | .026 | 219 | 82 |
|  | 220 | 33.000 | 1.00 | . | . | 220 | 81 |
|  | 221 | 33.000 | 1.00 | . | . | 221 | 80 |
|  | 222 | 33.000 | 1.00 | . | . | 222 | 79 |
|  | 223 | 33.000 | 1.00 | . | . | 223 | 78 |
|  | 224 | 33.000 | 1.00 | . | . | 224 | 77 |
|  | 225 | 33.000 | 1.00 | . | . | 225 | 76 |
|  | 226 | 33.000 | 1.00 | . | . | 226 | 75 |
|  | 227 | 33.000 | 1.00 | . | . | 227 | 74 |
|  | 228 | 33.000 | 1.00 | . | . | 228 | 73 |
|  | 229 | 33.000 | 1.00 | . | . | 229 | 72 |
|  | 230 | 33.000 | 1.00 | . | . | 230 | 71 |
|  | 231 | 33.000 | 1.00 | . | . | 231 | 70 |
|  | 232 | 33.000 | 1.00 | . | . | 232 | 69 |
|  | 233 | 33.000 | 1.00 | . | . | 233 | 68 |
|  | 234 | 33.000 | 1.00 | . | . | 234 | 67 |
|  | 235 | 33.000 | 1.00 | . | . | 235 | 66 |
|  | 236 | 33.000 | 1.00 | . | . | 236 | 65 |
|  | 237 | 33.000 | 1.00 | . | . | 237 | 64 |
|  | 238 | 33.000 | 1.00 | . | . | 238 | 63 |
|  | 239 | 33.000 | 1.00 | . | . | 239 | 62 |
|  | 240 | 33.000 | 1.00 | . | . | 240 | 61 |
|  | 241 | 33.000 | 1.00 | . | . | 241 | 60 |
|  | 242 | 33.000 | 1.00 | . | . | 242 | 59 |
|  | 243 | 33.000 | 1.00 | . | . | 243 | 58 |
|  | 244 | 33.000 | 1.00 | . | . | 244 | 57 |
|  | 245 | 33.000 | 1.00 | . | . | 245 | 56 |
|  | 246 | 33.000 | 1.00 | . | . | 246 | 55 |
|  | 247 | 33.000 | 1.00 | . | . | 247 | 54 |
|  | 248 | 33.000 | 1.00 | . | . | 248 | 53 |
|  | 249 | 33.000 | 1.00 | . | . | 249 | 52 |
|  | 250 | 33.000 | 1.00 | . | . | 250 | 51 |
|  | 251 | 33.000 | 1.00 | . | . | 251 | 50 |
|  | 252 | 33.000 | 1.00 | .163 | .021 | 252 | 49 |
|  | 253 | 36.000 | 1.00 | . | . | 253 | 48 |
|  | 254 | 36.000 | 1.00 | . | . | 254 | 47 |
|  | 255 | 36.000 | 1.00 | . | . | 255 | 46 |
|  | 256 | 36.000 | 1.00 | . | . | 256 | 45 |
|  | 257 | 36.000 | 1.00 | . | . | 257 | 44 |
|  | 258 | 36.000 | 1.00 | . | . | 258 | 43 |
|  | 259 | 36.000 | 1.00 | . | . | 259 | 42 |
|  | 260 | 36.000 | 1.00 | . | . | 260 | 41 |
|  | 261 | 36.000 | 1.00 | . | . | 261 | 40 |
|  | 262 | 36.000 | 1.00 | . | . | 262 | 39 |
|  | 263 | 36.000 | 1.00 | . | . | 263 | 38 |
|  | 264 | 36.000 | 1.00 | . | . | 264 | 37 |
|  | 265 | 36.000 | 1.00 | . | . | 265 | 36 |
|  | 266 | 36.000 | 1.00 | . | . | 266 | 35 |
|  | 267 | 36.000 | 1.00 | . | . | 267 | 34 |
|  | 268 | 36.000 | 1.00 | . | . | 268 | 33 |
|  | 269 | 36.000 | 1.00 | . | . | 269 | 32 |
|  | 270 | 36.000 | 1.00 | . | . | 270 | 31 |
|  | 271 | 36.000 | 1.00 | .100 | .017 | 271 | 30 |
|  | 272 | 39.000 | 1.00 | . | . | 272 | 29 |
|  | 273 | 39.000 | 1.00 | . | . | 273 | 28 |
|  | 274 | 39.000 | 1.00 | . | . | 274 | 27 |
|  | 275 | 39.000 | 1.00 | . | . | 275 | 26 |
|  | 276 | 39.000 | 1.00 | . | . | 276 | 25 |
|  | 277 | 39.000 | 1.00 | . | . | 277 | 24 |
|  | 278 | 39.000 | 1.00 | . | . | 278 | 23 |
|  | 279 | 39.000 | 1.00 | . | . | 279 | 22 |
|  | 280 | 39.000 | 1.00 | . | . | 280 | 21 |
|  | 281 | 39.000 | 1.00 | . | . | 281 | 20 |
|  | 282 | 39.000 | 1.00 | . | . | 282 | 19 |
|  | 283 | 39.000 | 1.00 | . | . | 283 | 18 |
|  | 284 | 39.000 | 1.00 | . | . | 284 | 17 |
|  | 285 | 39.000 | 1.00 | . | . | 285 | 16 |
|  | 286 | 39.000 | 1.00 | . | . | 286 | 15 |
|  | 287 | 39.000 | 1.00 | .047 | .012 | 287 | 14 |
|  | 288 | 42.000 | 1.00 | . | . | 288 | 13 |
|  | 289 | 42.000 | 1.00 | . | . | 289 | 12 |
|  | 290 | 42.000 | 1.00 | . | . | 290 | 11 |
|  | 291 | 42.000 | 1.00 | . | . | 291 | 10 |
|  | 292 | 42.000 | 1.00 | . | . | 292 | 9 |
|  | 293 | 42.000 | 1.00 | .027 | .009 | 293 | 8 |
|  | 294 | 45.000 | 1.00 | . | . | 294 | 7 |
|  | 295 | 45.000 | 1.00 | . | . | 295 | 6 |
|  | 296 | 45.000 | 1.00 | . | . | 296 | 5 |
|  | 297 | 45.000 | 1.00 | . | . | 297 | 4 |
|  | 298 | 45.000 | 1.00 | . | . | 298 | 3 |
|  | 299 | 45.000 | 1.00 | . | . | 299 | 2 |
|  | 300 | 45.000 | 1.00 | . | . | 300 | 1 |
|  | 301 | 45.000 | 1.00 | .000 | .000 | 301 | 0 |
| 2.00 | 1 | 9.000 | 1.00 | . | . | 1 | 341 |
|  | 2 | 9.000 | 1.00 | . | . | 2 | 340 |
|  | 3 | 9.000 | 1.00 | . | . | 3 | 339 |
|  | 4 | 9.000 | 1.00 | . | . | 4 | 338 |
|  | 5 | 9.000 | 1.00 | . | . | 5 | 337 |
|  | 6 | 9.000 | 1.00 | . | . | 6 | 336 |
|  | 7 | 9.000 | 1.00 | . | . | 7 | 335 |
|  | 8 | 9.000 | 1.00 | . | . | 8 | 334 |
|  | 9 | 9.000 | 1.00 | . | . | 9 | 333 |
|  | 10 | 9.000 | 1.00 | . | . | 10 | 332 |
|  | 11 | 9.000 | 1.00 | . | . | 11 | 331 |
|  | 12 | 9.000 | 1.00 | . | . | 12 | 330 |
|  | 13 | 9.000 | 1.00 | .962 | .010 | 13 | 329 |
|  | 14 | 12.000 | 1.00 | . | . | 14 | 328 |
|  | 15 | 12.000 | 1.00 | . | . | 15 | 327 |
|  | 16 | 12.000 | 1.00 | . | . | 16 | 326 |
|  | 17 | 12.000 | 1.00 | . | . | 17 | 325 |
|  | 18 | 12.000 | 1.00 | . | . | 18 | 324 |
|  | 19 | 12.000 | 1.00 | . | . | 19 | 323 |
|  | 20 | 12.000 | 1.00 | . | . | 20 | 322 |
|  | 21 | 12.000 | 1.00 | . | . | 21 | 321 |
|  | 22 | 12.000 | 1.00 | . | . | 22 | 320 |
|  | 23 | 12.000 | 1.00 | . | . | 23 | 319 |
|  | 24 | 12.000 | 1.00 | . | . | 24 | 318 |
|  | 25 | 12.000 | 1.00 | . | . | 25 | 317 |
|  | 26 | 12.000 | 1.00 | . | . | 26 | 316 |
|  | 27 | 12.000 | 1.00 | . | . | 27 | 315 |
|  | 28 | 12.000 | 1.00 | . | . | 28 | 314 |
|  | 29 | 12.000 | 1.00 | . | . | 29 | 313 |
|  | 30 | 12.000 | 1.00 | . | . | 30 | 312 |
|  | 31 | 12.000 | 1.00 | . | . | 31 | 311 |
|  | 32 | 12.000 | 1.00 | . | . | 32 | 310 |
|  | 33 | 12.000 | 1.00 | . | . | 33 | 309 |
|  | 34 | 12.000 | 1.00 | . | . | 34 | 308 |
|  | 35 | 12.000 | 1.00 | . | . | 35 | 307 |
|  | 36 | 12.000 | 1.00 | . | . | 36 | 306 |
|  | 37 | 12.000 | 1.00 | .892 | .017 | 37 | 305 |
|  | 38 | 15.000 | 1.00 | . | . | 38 | 304 |
|  | 39 | 15.000 | 1.00 | . | . | 39 | 303 |
|  | 40 | 15.000 | 1.00 | . | . | 40 | 302 |
|  | 41 | 15.000 | 1.00 | . | . | 41 | 301 |
|  | 42 | 15.000 | 1.00 | . | . | 42 | 300 |
|  | 43 | 15.000 | 1.00 | . | . | 43 | 299 |
|  | 44 | 15.000 | 1.00 | . | . | 44 | 298 |
|  | 45 | 15.000 | 1.00 | . | . | 45 | 297 |
|  | 46 | 15.000 | 1.00 | . | . | 46 | 296 |
|  | 47 | 15.000 | 1.00 | . | . | 47 | 295 |
|  | 48 | 15.000 | 1.00 | . | . | 48 | 294 |
|  | 49 | 15.000 | 1.00 | . | . | 49 | 293 |
|  | 50 | 15.000 | 1.00 | . | . | 50 | 292 |
|  | 51 | 15.000 | 1.00 | . | . | 51 | 291 |
|  | 52 | 15.000 | 1.00 | . | . | 52 | 290 |
|  | 53 | 15.000 | 1.00 | . | . | 53 | 289 |
|  | 54 | 15.000 | 1.00 | . | . | 54 | 288 |
|  | 55 | 15.000 | 1.00 | . | . | 55 | 287 |
|  | 56 | 15.000 | 1.00 | . | . | 56 | 286 |
|  | 57 | 15.000 | 1.00 | . | . | 57 | 285 |
|  | 58 | 15.000 | 1.00 | . | . | 58 | 284 |
|  | 59 | 15.000 | 1.00 | . | . | 59 | 283 |
|  | 60 | 15.000 | 1.00 | . | . | 60 | 282 |
|  | 61 | 15.000 | 1.00 | . | . | 61 | 281 |
|  | 62 | 15.000 | 1.00 | .819 | .021 | 62 | 280 |
|  | 63 | 18.000 | 1.00 | . | . | 63 | 279 |
|  | 64 | 18.000 | 1.00 | . | . | 64 | 278 |
|  | 65 | 18.000 | 1.00 | . | . | 65 | 277 |
|  | 66 | 18.000 | 1.00 | . | . | 66 | 276 |
|  | 67 | 18.000 | 1.00 | . | . | 67 | 275 |
|  | 68 | 18.000 | 1.00 | . | . | 68 | 274 |
|  | 69 | 18.000 | 1.00 | . | . | 69 | 273 |
|  | 70 | 18.000 | 1.00 | . | . | 70 | 272 |
|  | 71 | 18.000 | 1.00 | . | . | 71 | 271 |
|  | 72 | 18.000 | 1.00 | . | . | 72 | 270 |
|  | 73 | 18.000 | 1.00 | . | . | 73 | 269 |
|  | 74 | 18.000 | 1.00 | . | . | 74 | 268 |
|  | 75 | 18.000 | 1.00 | . | . | 75 | 267 |
|  | 76 | 18.000 | 1.00 | .778 | .022 | 76 | 266 |
|  | 77 | 21.000 | 1.00 | . | . | 77 | 265 |
|  | 78 | 21.000 | 1.00 | . | . | 78 | 264 |
|  | 79 | 21.000 | 1.00 | . | . | 79 | 263 |
|  | 80 | 21.000 | 1.00 | . | . | 80 | 262 |
|  | 81 | 21.000 | 1.00 | . | . | 81 | 261 |
|  | 82 | 21.000 | 1.00 | . | . | 82 | 260 |
|  | 83 | 21.000 | 1.00 | . | . | 83 | 259 |
|  | 84 | 21.000 | 1.00 | . | . | 84 | 258 |
|  | 85 | 21.000 | 1.00 | . | . | 85 | 257 |
|  | 86 | 21.000 | 1.00 | . | . | 86 | 256 |
|  | 87 | 21.000 | 1.00 | . | . | 87 | 255 |
|  | 88 | 21.000 | 1.00 | .743 | .024 | 88 | 254 |
|  | 89 | 24.000 | 1.00 | . | . | 89 | 253 |
|  | 90 | 24.000 | 1.00 | . | . | 90 | 252 |
|  | 91 | 24.000 | 1.00 | . | . | 91 | 251 |
|  | 92 | 24.000 | 1.00 | . | . | 92 | 250 |
|  | 93 | 24.000 | 1.00 | . | . | 93 | 249 |
|  | 94 | 24.000 | 1.00 | . | . | 94 | 248 |
|  | 95 | 24.000 | 1.00 | . | . | 95 | 247 |
|  | 96 | 24.000 | 1.00 | . | . | 96 | 246 |
|  | 97 | 24.000 | 1.00 | . | . | 97 | 245 |
|  | 98 | 24.000 | 1.00 | . | . | 98 | 244 |
|  | 99 | 24.000 | 1.00 | . | . | 99 | 243 |
|  | 100 | 24.000 | 1.00 | . | . | 100 | 242 |
|  | 101 | 24.000 | 1.00 | . | . | 101 | 241 |
|  | 102 | 24.000 | 1.00 | . | . | 102 | 240 |
|  | 103 | 24.000 | 1.00 | .699 | .025 | 103 | 239 |
|  | 104 | 27.000 | 1.00 | . | . | 104 | 238 |
|  | 105 | 27.000 | 1.00 | . | . | 105 | 237 |
|  | 106 | 27.000 | 1.00 | . | . | 106 | 236 |
|  | 107 | 27.000 | 1.00 | . | . | 107 | 235 |
|  | 108 | 27.000 | 1.00 | . | . | 108 | 234 |
|  | 109 | 27.000 | 1.00 | . | . | 109 | 233 |
|  | 110 | 27.000 | 1.00 | . | . | 110 | 232 |
|  | 111 | 27.000 | 1.00 | . | . | 111 | 231 |
|  | 112 | 27.000 | 1.00 | . | . | 112 | 230 |
|  | 113 | 27.000 | 1.00 | . | . | 113 | 229 |
|  | 114 | 27.000 | 1.00 | . | . | 114 | 228 |
|  | 115 | 27.000 | 1.00 | . | . | 115 | 227 |
|  | 116 | 27.000 | 1.00 | . | . | 116 | 226 |
|  | 117 | 27.000 | 1.00 | . | . | 117 | 225 |
|  | 118 | 27.000 | 1.00 | . | . | 118 | 224 |
|  | 119 | 27.000 | 1.00 | . | . | 119 | 223 |
|  | 120 | 27.000 | 1.00 | . | . | 120 | 222 |
|  | 121 | 27.000 | 1.00 | . | . | 121 | 221 |
|  | 122 | 27.000 | 1.00 | . | . | 122 | 220 |
|  | 123 | 27.000 | 1.00 | . | . | 123 | 219 |
|  | 124 | 27.000 | 1.00 | .637 | .026 | 124 | 218 |
|  | 125 | 30.000 | 1.00 | . | . | 125 | 217 |
|  | 126 | 30.000 | 1.00 | . | . | 126 | 216 |
|  | 127 | 30.000 | 1.00 | . | . | 127 | 215 |
|  | 128 | 30.000 | 1.00 | . | . | 128 | 214 |
|  | 129 | 30.000 | 1.00 | . | . | 129 | 213 |
|  | 130 | 30.000 | 1.00 | . | . | 130 | 212 |
|  | 131 | 30.000 | 1.00 | . | . | 131 | 211 |
|  | 132 | 30.000 | 1.00 | . | . | 132 | 210 |
|  | 133 | 30.000 | 1.00 | . | . | 133 | 209 |
|  | 134 | 30.000 | 1.00 | . | . | 134 | 208 |
|  | 135 | 30.000 | 1.00 | . | . | 135 | 207 |
|  | 136 | 30.000 | 1.00 | . | . | 136 | 206 |
|  | 137 | 30.000 | 1.00 | . | . | 137 | 205 |
|  | 138 | 30.000 | 1.00 | . | . | 138 | 204 |
|  | 139 | 30.000 | 1.00 | . | . | 139 | 203 |
|  | 140 | 30.000 | 1.00 | . | . | 140 | 202 |
|  | 141 | 30.000 | 1.00 | . | . | 141 | 201 |
|  | 142 | 30.000 | 1.00 | . | . | 142 | 200 |
|  | 143 | 30.000 | 1.00 | . | . | 143 | 199 |
|  | 144 | 30.000 | 1.00 | . | . | 144 | 198 |
|  | 145 | 30.000 | 1.00 | . | . | 145 | 197 |
|  | 146 | 30.000 | 1.00 | . | . | 146 | 196 |
|  | 147 | 30.000 | 1.00 | . | . | 147 | 195 |
|  | 148 | 30.000 | 1.00 | . | . | 148 | 194 |
|  | 149 | 30.000 | 1.00 | . | . | 149 | 193 |
|  | 150 | 30.000 | 1.00 | . | . | 150 | 192 |
|  | 151 | 30.000 | 1.00 | . | . | 151 | 191 |
|  | 152 | 30.000 | 1.00 | . | . | 152 | 190 |
|  | 153 | 30.000 | 1.00 | . | . | 153 | 189 |
|  | 154 | 30.000 | 1.00 | . | . | 154 | 188 |
|  | 155 | 30.000 | 1.00 | . | . | 155 | 187 |
|  | 156 | 30.000 | 1.00 | . | . | 156 | 186 |
|  | 157 | 30.000 | 1.00 | . | . | 157 | 185 |
|  | 158 | 30.000 | 1.00 | . | . | 158 | 184 |
|  | 159 | 30.000 | 1.00 | . | . | 159 | 183 |
|  | 160 | 30.000 | 1.00 | .532 | .027 | 160 | 182 |
|  | 161 | 33.000 | 1.00 | . | . | 161 | 181 |
|  | 162 | 33.000 | 1.00 | . | . | 162 | 180 |
|  | 163 | 33.000 | 1.00 | . | . | 163 | 179 |
|  | 164 | 33.000 | 1.00 | . | . | 164 | 178 |
|  | 165 | 33.000 | 1.00 | . | . | 165 | 177 |
|  | 166 | 33.000 | 1.00 | . | . | 166 | 176 |
|  | 167 | 33.000 | 1.00 | . | . | 167 | 175 |
|  | 168 | 33.000 | 1.00 | . | . | 168 | 174 |
|  | 169 | 33.000 | 1.00 | . | . | 169 | 173 |
|  | 170 | 33.000 | 1.00 | . | . | 170 | 172 |
|  | 171 | 33.000 | 1.00 | . | . | 171 | 171 |
|  | 172 | 33.000 | 1.00 | . | . | 172 | 170 |
|  | 173 | 33.000 | 1.00 | . | . | 173 | 169 |
|  | 174 | 33.000 | 1.00 | . | . | 174 | 168 |
|  | 175 | 33.000 | 1.00 | .488 | .027 | 175 | 167 |
|  | 176 | 36.000 | 1.00 | . | . | 176 | 166 |
|  | 177 | 36.000 | 1.00 | . | . | 177 | 165 |
|  | 178 | 36.000 | 1.00 | . | . | 178 | 164 |
|  | 179 | 36.000 | 1.00 | . | . | 179 | 163 |
|  | 180 | 36.000 | 1.00 | . | . | 180 | 162 |
|  | 181 | 36.000 | 1.00 | . | . | 181 | 161 |
|  | 182 | 36.000 | 1.00 | . | . | 182 | 160 |
|  | 183 | 36.000 | 1.00 | . | . | 183 | 159 |
|  | 184 | 36.000 | 1.00 | . | . | 184 | 158 |
|  | 185 | 36.000 | 1.00 | . | . | 185 | 157 |
|  | 186 | 36.000 | 1.00 | . | . | 186 | 156 |
|  | 187 | 36.000 | 1.00 | . | . | 187 | 155 |
|  | 188 | 36.000 | 1.00 | . | . | 188 | 154 |
|  | 189 | 36.000 | 1.00 | . | . | 189 | 153 |
|  | 190 | 36.000 | 1.00 | . | . | 190 | 152 |
|  | 191 | 36.000 | 1.00 | . | . | 191 | 151 |
|  | 192 | 36.000 | 1.00 | . | . | 192 | 150 |
|  | 193 | 36.000 | 1.00 | . | . | 193 | 149 |
|  | 194 | 36.000 | 1.00 | . | . | 194 | 148 |
|  | 195 | 36.000 | 1.00 | . | . | 195 | 147 |
|  | 196 | 36.000 | 1.00 | . | . | 196 | 146 |
|  | 197 | 36.000 | 1.00 | . | . | 197 | 145 |
|  | 198 | 36.000 | 1.00 | . | . | 198 | 144 |
|  | 199 | 36.000 | 1.00 | . | . | 199 | 143 |
|  | 200 | 36.000 | 1.00 | . | . | 200 | 142 |
|  | 201 | 36.000 | 1.00 | . | . | 201 | 141 |
|  | 202 | 36.000 | 1.00 | . | . | 202 | 140 |
|  | 203 | 36.000 | 1.00 | . | . | 203 | 139 |
|  | 204 | 36.000 | 1.00 | . | . | 204 | 138 |
|  | 205 | 36.000 | 1.00 | . | . | 205 | 137 |
|  | 206 | 36.000 | 1.00 | . | . | 206 | 136 |
|  | 207 | 36.000 | 1.00 | . | . | 207 | 135 |
|  | 208 | 36.000 | 1.00 | . | . | 208 | 134 |
|  | 209 | 36.000 | 1.00 | . | . | 209 | 133 |
|  | 210 | 36.000 | 1.00 | . | . | 210 | 132 |
|  | 211 | 36.000 | 1.00 | . | . | 211 | 131 |
|  | 212 | 36.000 | 1.00 | . | . | 212 | 130 |
|  | 213 | 36.000 | 1.00 | . | . | 213 | 129 |
|  | 214 | 36.000 | 1.00 | . | . | 214 | 128 |
|  | 215 | 36.000 | 1.00 | . | . | 215 | 127 |
|  | 216 | 36.000 | 1.00 | . | . | 216 | 126 |
|  | 217 | 36.000 | 1.00 | . | . | 217 | 125 |
|  | 218 | 36.000 | 1.00 | . | . | 218 | 124 |
|  | 219 | 36.000 | 1.00 | . | . | 219 | 123 |
|  | 220 | 36.000 | 1.00 | .357 | .026 | 220 | 122 |
|  | 221 | 39.000 | 1.00 | . | . | 221 | 121 |
|  | 222 | 39.000 | 1.00 | . | . | 222 | 120 |
|  | 223 | 39.000 | 1.00 | . | . | 223 | 119 |
|  | 224 | 39.000 | 1.00 | . | . | 224 | 118 |
|  | 225 | 39.000 | 1.00 | . | . | 225 | 117 |
|  | 226 | 39.000 | 1.00 | . | . | 226 | 116 |
|  | 227 | 39.000 | 1.00 | . | . | 227 | 115 |
|  | 228 | 39.000 | 1.00 | . | . | 228 | 114 |
|  | 229 | 39.000 | 1.00 | . | . | 229 | 113 |
|  | 230 | 39.000 | 1.00 | . | . | 230 | 112 |
|  | 231 | 39.000 | 1.00 | . | . | 231 | 111 |
|  | 232 | 39.000 | 1.00 | . | . | 232 | 110 |
|  | 233 | 39.000 | 1.00 | . | . | 233 | 109 |
|  | 234 | 39.000 | 1.00 | . | . | 234 | 108 |
|  | 235 | 39.000 | 1.00 | . | . | 235 | 107 |
|  | 236 | 39.000 | 1.00 | . | . | 236 | 106 |
|  | 237 | 39.000 | 1.00 | . | . | 237 | 105 |
|  | 238 | 39.000 | 1.00 | . | . | 238 | 104 |
|  | 239 | 39.000 | 1.00 | . | . | 239 | 103 |
|  | 240 | 39.000 | 1.00 | . | . | 240 | 102 |
|  | 241 | 39.000 | 1.00 | . | . | 241 | 101 |
|  | 242 | 39.000 | 1.00 | . | . | 242 | 100 |
|  | 243 | 39.000 | 1.00 | . | . | 243 | 99 |
|  | 244 | 39.000 | 1.00 | . | . | 244 | 98 |
|  | 245 | 39.000 | 1.00 | . | . | 245 | 97 |
|  | 246 | 39.000 | 1.00 | . | . | 246 | 96 |
|  | 247 | 39.000 | 1.00 | . | . | 247 | 95 |
|  | 248 | 39.000 | 1.00 | . | . | 248 | 94 |
|  | 249 | 39.000 | 1.00 | . | . | 249 | 93 |
|  | 250 | 39.000 | 1.00 | . | . | 250 | 92 |
|  | 251 | 39.000 | 1.00 | . | . | 251 | 91 |
|  | 252 | 39.000 | 1.00 | . | . | 252 | 90 |
|  | 253 | 39.000 | 1.00 | .260 | .024 | 253 | 89 |
|  | 254 | 42.000 | 1.00 | . | . | 254 | 88 |
|  | 255 | 42.000 | 1.00 | . | . | 255 | 87 |
|  | 256 | 42.000 | 1.00 | . | . | 256 | 86 |
|  | 257 | 42.000 | 1.00 | . | . | 257 | 85 |
|  | 258 | 42.000 | 1.00 | . | . | 258 | 84 |
|  | 259 | 42.000 | 1.00 | . | . | 259 | 83 |
|  | 260 | 42.000 | 1.00 | . | . | 260 | 82 |
|  | 261 | 42.000 | 1.00 | . | . | 261 | 81 |
|  | 262 | 42.000 | 1.00 | . | . | 262 | 80 |
|  | 263 | 42.000 | 1.00 | . | . | 263 | 79 |
|  | 264 | 42.000 | 1.00 | . | . | 264 | 78 |
|  | 265 | 42.000 | 1.00 | . | . | 265 | 77 |
|  | 266 | 42.000 | 1.00 | . | . | 266 | 76 |
|  | 267 | 42.000 | 1.00 | . | . | 267 | 75 |
|  | 268 | 42.000 | 1.00 | . | . | 268 | 74 |
|  | 269 | 42.000 | 1.00 | . | . | 269 | 73 |
|  | 270 | 42.000 | 1.00 | . | . | 270 | 72 |
|  | 271 | 42.000 | 1.00 | . | . | 271 | 71 |
|  | 272 | 42.000 | 1.00 | . | . | 272 | 70 |
|  | 273 | 42.000 | 1.00 | . | . | 273 | 69 |
|  | 274 | 42.000 | 1.00 | . | . | 274 | 68 |
|  | 275 | 42.000 | 1.00 | . | . | 275 | 67 |
|  | 276 | 42.000 | 1.00 | . | . | 276 | 66 |
|  | 277 | 42.000 | 1.00 | . | . | 277 | 65 |
|  | 278 | 42.000 | 1.00 | . | . | 278 | 64 |
|  | 279 | 42.000 | 1.00 | . | . | 279 | 63 |
|  | 280 | 42.000 | 1.00 | . | . | 280 | 62 |
|  | 281 | 42.000 | 1.00 | . | . | 281 | 61 |
|  | 282 | 42.000 | 1.00 | . | . | 282 | 60 |
|  | 283 | 42.000 | 1.00 | . | . | 283 | 59 |
|  | 284 | 42.000 | 1.00 | . | . | 284 | 58 |
|  | 285 | 42.000 | 1.00 | . | . | 285 | 57 |
|  | 286 | 42.000 | 1.00 | . | . | 286 | 56 |
|  | 287 | 42.000 | 1.00 | . | . | 287 | 55 |
|  | 288 | 42.000 | 1.00 | . | . | 288 | 54 |
|  | 289 | 42.000 | 1.00 | . | . | 289 | 53 |
|  | 290 | 42.000 | 1.00 | . | . | 290 | 52 |
|  | 291 | 42.000 | 1.00 | . | . | 291 | 51 |
|  | 292 | 42.000 | 1.00 | . | . | 292 | 50 |
|  | 293 | 42.000 | 1.00 | . | . | 293 | 49 |
|  | 294 | 42.000 | 1.00 | .140 | .019 | 294 | 48 |
|  | 295 | 45.000 | 1.00 | . | . | 295 | 47 |
|  | 296 | 45.000 | 1.00 | . | . | 296 | 46 |
|  | 297 | 45.000 | 1.00 | . | . | 297 | 45 |
|  | 298 | 45.000 | 1.00 | . | . | 298 | 44 |
|  | 299 | 45.000 | 1.00 | . | . | 299 | 43 |
|  | 300 | 45.000 | 1.00 | . | . | 300 | 42 |
|  | 301 | 45.000 | 1.00 | . | . | 301 | 41 |
|  | 302 | 45.000 | 1.00 | . | . | 302 | 40 |
|  | 303 | 45.000 | 1.00 | . | . | 303 | 39 |
|  | 304 | 45.000 | 1.00 | . | . | 304 | 38 |
|  | 305 | 45.000 | 1.00 | . | . | 305 | 37 |
|  | 306 | 45.000 | 1.00 | . | . | 306 | 36 |
|  | 307 | 45.000 | 1.00 | . | . | 307 | 35 |
|  | 308 | 45.000 | 1.00 | . | . | 308 | 34 |
|  | 309 | 45.000 | 1.00 | . | . | 309 | 33 |
|  | 310 | 45.000 | 1.00 | . | . | 310 | 32 |
|  | 311 | 45.000 | 1.00 | . | . | 311 | 31 |
|  | 312 | 45.000 | 1.00 | . | . | 312 | 30 |
|  | 313 | 45.000 | 1.00 | . | . | 313 | 29 |
|  | 314 | 45.000 | 1.00 | . | . | 314 | 28 |
|  | 315 | 45.000 | 1.00 | . | . | 315 | 27 |
|  | 316 | 45.000 | 1.00 | . | . | 316 | 26 |
|  | 317 | 45.000 | 1.00 | . | . | 317 | 25 |
|  | 318 | 45.000 | 1.00 | . | . | 318 | 24 |
|  | 319 | 45.000 | 1.00 | . | . | 319 | 23 |
|  | 320 | 45.000 | 1.00 | . | . | 320 | 22 |
|  | 321 | 45.000 | 1.00 | . | . | 321 | 21 |
|  | 322 | 45.000 | 1.00 | . | . | 322 | 20 |
|  | 323 | 45.000 | 1.00 | . | . | 323 | 19 |
|  | 324 | 45.000 | 1.00 | . | . | 324 | 18 |
|  | 325 | 45.000 | 1.00 | . | . | 325 | 17 |
|  | 326 | 45.000 | 1.00 | . | . | 326 | 16 |
|  | 327 | 45.000 | 1.00 | .044 | .011 | 327 | 15 |
|  | 328 | 48.000 | 1.00 | . | . | 328 | 14 |
|  | 329 | 48.000 | 1.00 | . | . | 329 | 13 |
|  | 330 | 48.000 | 1.00 | . | . | 330 | 12 |
|  | 331 | 48.000 | 1.00 | . | . | 331 | 11 |
|  | 332 | 48.000 | 1.00 | . | . | 332 | 10 |
|  | 333 | 48.000 | 1.00 | . | . | 333 | 9 |
|  | 334 | 48.000 | 1.00 | . | . | 334 | 8 |
|  | 335 | 48.000 | 1.00 | . | . | 335 | 7 |
|  | 336 | 48.000 | 1.00 | . | . | 336 | 6 |
|  | 337 | 48.000 | 1.00 | . | . | 337 | 5 |
|  | 338 | 48.000 | 1.00 | . | . | 338 | 4 |
|  | 339 | 48.000 | 1.00 | . | . | 339 | 3 |
|  | 340 | 48.000 | 1.00 | . | . | 340 | 2 |
|  | 341 | 48.000 | 1.00 | . | . | 341 | 1 |
|  | 342 | 48.000 | 1.00 | .000 | .000 | 342 | 0 |

| **Means and Medians for Survival Time** | | | | | | | | |
| --- | --- | --- | --- | --- | --- | --- | --- | --- |
| group | Mean^a^ | | | | Median | | | |
|  |  | | 95% Confidence Interval | |  | | 95% Confidence Interval | |
|  | Estimate | Std. Error | Lower Bound | Upper Bound | Estimate | Std. Error | Lower Bound | Upper Bound |
| 1.00 | 26.183 | .512 | 25.180 | 27.186 | 27.000 | .486 | 26.048 | 27.952 |
| 2.00 | 31.053 | .622 | 29.834 | 32.271 | 33.000 | .867 | 31.301 | 34.699 |
| Overall | 28.773 | .419 | 27.951 | 29.595 | 30.000 | .480 | 29.060 | 30.940 |
| a. Estimation is limited to the largest survival time if it is censored. | | | | | | | | |
