# Supplementary material for: rBmαTX14 Increases the Life Span and Promotes the Locomotion of Caenorhabditis Elegans
Source: PLoS One. 2016 Sep 9;11(9):e0161847. doi: 10.1371/journal.pone.0161847 (PMC5017660; doi:10.1371/journal.pone.0161847)
Supplement: S2 Table — Gene lists were for 171 Up and 11 Down regulation genes. (DOC) [file pone.0161847.s003.doc]

**S2 Table. The different genes in *C. elegans* fed with *rBmα*TX14 through Affymetrix Microarray analysis.** Gene lists were for 171 Up and 11 Down regulation genes.

171 Up-regulated genes

| R12E2.14 | F44E2.4 | ZK1025.3 | COL-130 |
| --- | --- | --- | --- |
| PTR-23 | Y47D7A.13 | PES-8 | COL-126/COL-127 |
| CLEC-174 | Y22D7AL.14 | MLT-8 | NAS-27 |
| F10D11.6 | Y47D7A.13 | Y11D7A.9 | COL-60 |
| ABU-7 | F35B3.4 | Y47D3B.6 | COL-97 |
| ABU-6 | COL-90 | C06G1.1 | TYR-2 |
| C02E7.7 | COL-91 | R02E4.1 | F58E6.13/EGF-like domain |
| NSPB-12 | ABU-1/PQN-2 | ZK180.5 | NAS-30 |
| R12E2.7 | AGMO-1 | F45E4.5 | COL-48 |
| C02E7.6 | DAO-4 | PQN-54 | COL-77 |
| ABU-15 | C05E7.2 | ABU-14 | locus:col-175 |
| PQN-90 | C30H6.5 | COL-138 | EFL-3 |
| PQN-91 | E01G4.6 | C05E7.1 | COL-49 |
| NSPB-10 | C06A1.6 | NLP-25 | COL-104 |
| COL-145 | F33A8.7 | T10E9.3 | COL-38 |
| R12E2.15 | T23F6.1 | ABU-13 | COL-88 |
| W08E12.3 | C34E7.4 | TRX-3 | SURO-1 |
| W08E12.4/W08E12.5 | GRL-21 | ZC204.12 | WRT-4 |
| R12E2.14 | LYS-6 | F58E10.7 | DPY-5 |
| PQN-76/PQN-78/PQN-79/ PQN-91 | R03C1.1 | C46H11.2 | OV-17 antigen precursor/ZK970.7 |
| ABU-8 | T03F6.4 | Y53F4B.27 | NAS-7 |
| ABU-5/PQN-76/PQN-78/PQN-79/PQN-90/PQN-91 | PQN-71 | F52F10.2 | H10E21.4 |
| Y105C5A.12/Y105C5A.13 | LGC-21 | R02F11.1 | UGT-6 |
| F53F1.4 | ZK662.2 | Y95B8A.2 | " Y41C4A.11/WD domain, G-beta repeat" |
| F41F3.3 | PQN-74 | F33D4.6 | C54D10.10 |
| CUT-2 | B0024.4 | F41E6.11 | F15B9.8 |
| COL-107 | TAG-297 | H23N18.5 | FMO-1 |
| SURO-1 | K04C2.5 | GRD-14 | COL-14 |
| GRL-7 | ABU-10 | ABU-9/PQN-57 | HMIT-1.1 |
| H10E21.4 | COL-137 | F57H12.6 | M153.1 |
| COL-109 | DAO-2/M03A1.8 | Y51H7C.13 | T28H10.3 |
| ABU-11 | DAO-2 | GRL-16 | BUS-19 |
| COL-113 | C26B9.3 | COL-71 | SQT-1 |
| ZK180.5 | PQN-32 | D2096.6 | ROL-6 |
| COL-120 | K10H10.4 | DCT-5 | NNT-1 |
| TTR-44 | T22B2.6 | T25E4.1 | C06E8.5 |
| ABU-14 | C42D4.3 | COL-156 | F32D8.7 |
| ncRNA | T17H7.7 | LEA-1 | FIPR-26 |
| M03F4.6 | LRON-2 | locus:grd-6 | LPR-3 |
| Y18H1A.9 | PQN-63 | WRT-6 | ABU-8 |
| LEA-1 | F26G1.5 | PTR-23 | F44E2.4 |
| T19A5.3 | F10D11.6 | PTR-23 | T05F1.11 |
| SQT-3 | T19B10.2 | COL-161/COL-162 | ROL-1 |
| COL-97 | W03F8.6 | RAM-2 |  |

11 Down-regulated genes

| W03F11.1 | SEA-1 | ncRNA/ncRNA | COL-101 |
| --- | --- | --- | --- |
| CAV-1 | COL-143 | VIT-1 | ASM-3 |
| T22B7.7 | F26A1.8 | " T01G5.7/Zinc finger, C3HC4 type (RING finger)" |  |
