# Supplementary material for: rBmαTX14 Increases the Life Span and Promotes the Locomotion of Caenorhabditis Elegans
Source: PLoS One. 2016 Sep 9;11(9):e0161847. doi: 10.1371/journal.pone.0161847 (PMC5017660; doi:10.1371/journal.pone.0161847)
Supplement: S3 Table — Group 1 is C. elegans fed with control E.coli strain with empty vector, Group 2 is C. elegans fed with pET28a-rBmαTX14, Group 3 is C. elegans fed with pET28a-rBmαTX14 (H15⟶F15), and Group 4 is C. elegans fed with pET28a-rBmαTX14 (T18⟶R18). (DOC) [file pone.0161847.s004.doc]

**S3 Table. Amino acid mutations of r*Bm*αTX14 affect the life span extension of *C. elegans.***

**Kaplan-Meier**

| **Notes** | | |
| --- | --- | --- |
|  | Output Created | 28-1-2013 |
|  | Comments |  |
| Input | Active Dataset | data0 |
|  | Filter | <none> |
|  | Weight | <none> |
|  | Split File | <none> |
|  | N of Rows in Working Data File | 618 |
| Missing Value Handling | Definition of Missing | User-defined missing values are treated as missing. |
|  | Cases Used | Statistics are based on all cases with valid data for all variables in the analysis. |
|  | Syntax | KM time BY group  /STATUS=state(1)  /PRINT TABLE MEAN  /PLOT SURVIVAL  /TEST LOGRANK  /COMPARE PAIRWISE POOLED  /SAVE SURVIVAL SE. |
| Resources | Processor Time | 0:00:01.045 |
|  | Elapsed Time | 0:00:01.075 |
| Variables Created or Modified | SUR_1 | Survival function |
|  | SE_1 | Standard error of the survival function |

[data0]

| **Case Processing Summary** | | | | |
| --- | --- | --- | --- | --- |
| group |  | | Censored | |
|  | Total N | N of Events | N | Percent |
| 1.00 | 148 | 148 | 0 | .0% |
| 2.00 | 166 | 166 | 0 | .0% |
| 3.00 | 152 | 152 | 0 | .0% |
| 4.00 | 149 | 149 | 0 | .0% |
| Overall | 615 | 615 | 0 | .0% |

| **Survival Table** | | | | | | | |
| --- | --- | --- | --- | --- | --- | --- | --- |
| group | |  | | Cumulative Proportion Surviving at the Time | |  | |
|  |  | Time | Status | Estimate | Std. Error | N of Cumulative Events | N of Remaining Cases |
| 1.00 | 1 | 9.000 | 1.00 | . | . | 1 | 147 |
|  | 2 | 9.000 | 1.00 | . | . | 2 | 146 |
|  | 3 | 9.000 | 1.00 | . | . | 3 | 145 |
|  | 4 | 9.000 | 1.00 | . | . | 4 | 144 |
|  | 5 | 9.000 | 1.00 | . | . | 5 | 143 |
|  | 6 | 9.000 | 1.00 | . | . | 6 | 142 |
|  | 7 | 9.000 | 1.00 | . | . | 7 | 141 |
|  | 8 | 9.000 | 1.00 | . | . | 8 | 140 |
|  | 9 | 9.000 | 1.00 | .939 | .020 | 9 | 139 |
|  | 10 | 12.000 | 1.00 | . | . | 10 | 138 |
|  | 11 | 12.000 | 1.00 | . | . | 11 | 137 |
|  | 12 | 12.000 | 1.00 | . | . | 12 | 136 |
|  | 13 | 12.000 | 1.00 | . | . | 13 | 135 |
|  | 14 | 12.000 | 1.00 | . | . | 14 | 134 |
|  | 15 | 12.000 | 1.00 | . | . | 15 | 133 |
|  | 16 | 12.000 | 1.00 | . | . | 16 | 132 |
|  | 17 | 12.000 | 1.00 | . | . | 17 | 131 |
|  | 18 | 12.000 | 1.00 | . | . | 18 | 130 |
|  | 19 | 12.000 | 1.00 | .872 | .027 | 19 | 129 |
|  | 20 | 15.000 | 1.00 | . | . | 20 | 128 |
|  | 21 | 15.000 | 1.00 | . | . | 21 | 127 |
|  | 22 | 15.000 | 1.00 | . | . | 22 | 126 |
|  | 23 | 15.000 | 1.00 | .845 | .030 | 23 | 125 |
|  | 24 | 18.000 | 1.00 | . | . | 24 | 124 |
|  | 25 | 18.000 | 1.00 | . | . | 25 | 123 |
|  | 26 | 18.000 | 1.00 | . | . | 26 | 122 |
|  | 27 | 18.000 | 1.00 | . | . | 27 | 121 |
|  | 28 | 18.000 | 1.00 | . | . | 28 | 120 |
|  | 29 | 18.000 | 1.00 | . | . | 29 | 119 |
|  | 30 | 18.000 | 1.00 | .797 | .033 | 30 | 118 |
|  | 31 | 21.000 | 1.00 | . | . | 31 | 117 |
|  | 32 | 21.000 | 1.00 | . | . | 32 | 116 |
|  | 33 | 21.000 | 1.00 | . | . | 33 | 115 |
|  | 34 | 21.000 | 1.00 | . | . | 34 | 114 |
|  | 35 | 21.000 | 1.00 | . | . | 35 | 113 |
|  | 36 | 21.000 | 1.00 | . | . | 36 | 112 |
|  | 37 | 21.000 | 1.00 | . | . | 37 | 111 |
|  | 38 | 21.000 | 1.00 | . | . | 38 | 110 |
|  | 39 | 21.000 | 1.00 | . | . | 39 | 109 |
|  | 40 | 21.000 | 1.00 | . | . | 40 | 108 |
|  | 41 | 21.000 | 1.00 | . | . | 41 | 107 |
|  | 42 | 21.000 | 1.00 | . | . | 42 | 106 |
|  | 43 | 21.000 | 1.00 | . | . | 43 | 105 |
|  | 44 | 21.000 | 1.00 | . | . | 44 | 104 |
|  | 45 | 21.000 | 1.00 | . | . | 45 | 103 |
|  | 46 | 21.000 | 1.00 | . | . | 46 | 102 |
|  | 47 | 21.000 | 1.00 | . | . | 47 | 101 |
|  | 48 | 21.000 | 1.00 | . | . | 48 | 100 |
|  | 49 | 21.000 | 1.00 | . | . | 49 | 99 |
|  | 50 | 21.000 | 1.00 | .662 | .039 | 50 | 98 |
|  | 51 | 24.000 | 1.00 | . | . | 51 | 97 |
|  | 52 | 24.000 | 1.00 | . | . | 52 | 96 |
|  | 53 | 24.000 | 1.00 | . | . | 53 | 95 |
|  | 54 | 24.000 | 1.00 | . | . | 54 | 94 |
|  | 55 | 24.000 | 1.00 | . | . | 55 | 93 |
|  | 56 | 24.000 | 1.00 | . | . | 56 | 92 |
|  | 57 | 24.000 | 1.00 | . | . | 57 | 91 |
|  | 58 | 24.000 | 1.00 | . | . | 58 | 90 |
|  | 59 | 24.000 | 1.00 | . | . | 59 | 89 |
|  | 60 | 24.000 | 1.00 | . | . | 60 | 88 |
|  | 61 | 24.000 | 1.00 | . | . | 61 | 87 |
|  | 62 | 24.000 | 1.00 | . | . | 62 | 86 |
|  | 63 | 24.000 | 1.00 | . | . | 63 | 85 |
|  | 64 | 24.000 | 1.00 | . | . | 64 | 84 |
|  | 65 | 24.000 | 1.00 | .561 | .041 | 65 | 83 |
|  | 66 | 27.000 | 1.00 | . | . | 66 | 82 |
|  | 67 | 27.000 | 1.00 | . | . | 67 | 81 |
|  | 68 | 27.000 | 1.00 | . | . | 68 | 80 |
|  | 69 | 27.000 | 1.00 | . | . | 69 | 79 |
|  | 70 | 27.000 | 1.00 | . | . | 70 | 78 |
|  | 71 | 27.000 | 1.00 | . | . | 71 | 77 |
|  | 72 | 27.000 | 1.00 | . | . | 72 | 76 |
|  | 73 | 27.000 | 1.00 | . | . | 73 | 75 |
|  | 74 | 27.000 | 1.00 | . | . | 74 | 74 |
|  | 75 | 27.000 | 1.00 | . | . | 75 | 73 |
|  | 76 | 27.000 | 1.00 | . | . | 76 | 72 |
|  | 77 | 27.000 | 1.00 | . | . | 77 | 71 |
|  | 78 | 27.000 | 1.00 | . | . | 78 | 70 |
|  | 79 | 27.000 | 1.00 | . | . | 79 | 69 |
|  | 80 | 27.000 | 1.00 | .459 | .041 | 80 | 68 |
|  | 81 | 31.000 | 1.00 | . | . | 81 | 67 |
|  | 82 | 31.000 | 1.00 | . | . | 82 | 66 |
|  | 83 | 31.000 | 1.00 | . | . | 83 | 65 |
|  | 84 | 31.000 | 1.00 | . | . | 84 | 64 |
|  | 85 | 31.000 | 1.00 | . | . | 85 | 63 |
|  | 86 | 31.000 | 1.00 | . | . | 86 | 62 |
|  | 87 | 31.000 | 1.00 | . | . | 87 | 61 |
|  | 88 | 31.000 | 1.00 | . | . | 88 | 60 |
|  | 89 | 31.000 | 1.00 | . | . | 89 | 59 |
|  | 90 | 31.000 | 1.00 | . | . | 90 | 58 |
|  | 91 | 31.000 | 1.00 | . | . | 91 | 57 |
|  | 92 | 31.000 | 1.00 | . | . | 92 | 56 |
|  | 93 | 31.000 | 1.00 | . | . | 93 | 55 |
|  | 94 | 31.000 | 1.00 | . | . | 94 | 54 |
|  | 95 | 31.000 | 1.00 | . | . | 95 | 53 |
|  | 96 | 31.000 | 1.00 | . | . | 96 | 52 |
|  | 97 | 31.000 | 1.00 | . | . | 97 | 51 |
|  | 98 | 31.000 | 1.00 | . | . | 98 | 50 |
|  | 99 | 31.000 | 1.00 | . | . | 99 | 49 |
|  | 100 | 31.000 | 1.00 | . | . | 100 | 48 |
|  | 101 | 31.000 | 1.00 | . | . | 101 | 47 |
|  | 102 | 31.000 | 1.00 | . | . | 102 | 46 |
|  | 103 | 31.000 | 1.00 | . | . | 103 | 45 |
|  | 104 | 31.000 | 1.00 | . | . | 104 | 44 |
|  | 105 | 31.000 | 1.00 | . | . | 105 | 43 |
|  | 106 | 31.000 | 1.00 | . | . | 106 | 42 |
|  | 107 | 31.000 | 1.00 | . | . | 107 | 41 |
|  | 108 | 31.000 | 1.00 | . | . | 108 | 40 |
|  | 109 | 31.000 | 1.00 | . | . | 109 | 39 |
|  | 110 | 31.000 | 1.00 | . | . | 110 | 38 |
|  | 111 | 31.000 | 1.00 | .250 | .036 | 111 | 37 |
|  | 112 | 34.000 | 1.00 | . | . | 112 | 36 |
|  | 113 | 34.000 | 1.00 | . | . | 113 | 35 |
|  | 114 | 34.000 | 1.00 | . | . | 114 | 34 |
|  | 115 | 34.000 | 1.00 | . | . | 115 | 33 |
|  | 116 | 34.000 | 1.00 | . | . | 116 | 32 |
|  | 117 | 34.000 | 1.00 | . | . | 117 | 31 |
|  | 118 | 34.000 | 1.00 | . | . | 118 | 30 |
|  | 119 | 34.000 | 1.00 | . | . | 119 | 29 |
|  | 120 | 34.000 | 1.00 | . | . | 120 | 28 |
|  | 121 | 34.000 | 1.00 | . | . | 121 | 27 |
|  | 122 | 34.000 | 1.00 | . | . | 122 | 26 |
|  | 123 | 34.000 | 1.00 | . | . | 123 | 25 |
|  | 124 | 34.000 | 1.00 | . | . | 124 | 24 |
|  | 125 | 34.000 | 1.00 | . | . | 125 | 23 |
|  | 126 | 34.000 | 1.00 | .149 | .029 | 126 | 22 |
|  | 127 | 37.000 | 1.00 | . | . | 127 | 21 |
|  | 128 | 37.000 | 1.00 | . | . | 128 | 20 |
|  | 129 | 37.000 | 1.00 | . | . | 129 | 19 |
|  | 130 | 37.000 | 1.00 | . | . | 130 | 18 |
|  | 131 | 37.000 | 1.00 | . | . | 131 | 17 |
|  | 132 | 37.000 | 1.00 | . | . | 132 | 16 |
|  | 133 | 37.000 | 1.00 | . | . | 133 | 15 |
|  | 134 | 37.000 | 1.00 | . | . | 134 | 14 |
|  | 135 | 37.000 | 1.00 | . | . | 135 | 13 |
|  | 136 | 37.000 | 1.00 | . | . | 136 | 12 |
|  | 137 | 37.000 | 1.00 | . | . | 137 | 11 |
|  | 138 | 37.000 | 1.00 | . | . | 138 | 10 |
|  | 139 | 37.000 | 1.00 | . | . | 139 | 9 |
|  | 140 | 37.000 | 1.00 | . | . | 140 | 8 |
|  | 141 | 37.000 | 1.00 | .047 | .017 | 141 | 7 |
|  | 142 | 41.000 | 1.00 | . | . | 142 | 6 |
|  | 143 | 41.000 | 1.00 | . | . | 143 | 5 |
|  | 144 | 41.000 | 1.00 | . | . | 144 | 4 |
|  | 145 | 41.000 | 1.00 | . | . | 145 | 3 |
|  | 146 | 41.000 | 1.00 | . | . | 146 | 2 |
|  | 147 | 41.000 | 1.00 | .007 | .007 | 147 | 1 |
|  | 148 | 44.000 | 1.00 | .000 | .000 | 148 | 0 |
| 2.00 | 1 | 6.000 | 1.00 | . | . | 1 | 165 |
|  | 2 | 6.000 | 1.00 | .988 | .008 | 2 | 164 |
|  | 3 | 9.000 | 1.00 | . | . | 3 | 163 |
|  | 4 | 9.000 | 1.00 | . | . | 4 | 162 |
|  | 5 | 9.000 | 1.00 | . | . | 5 | 161 |
|  | 6 | 9.000 | 1.00 | . | . | 6 | 160 |
|  | 7 | 9.000 | 1.00 | . | . | 7 | 159 |
|  | 8 | 9.000 | 1.00 | . | . | 8 | 158 |
|  | 9 | 9.000 | 1.00 | . | . | 9 | 157 |
|  | 10 | 9.000 | 1.00 | . | . | 10 | 156 |
|  | 11 | 9.000 | 1.00 | . | . | 11 | 155 |
|  | 12 | 9.000 | 1.00 | . | . | 12 | 154 |
|  | 13 | 9.000 | 1.00 | . | . | 13 | 153 |
|  | 14 | 9.000 | 1.00 | .916 | .022 | 14 | 152 |
|  | 15 | 12.000 | 1.00 | . | . | 15 | 151 |
|  | 16 | 12.000 | 1.00 | . | . | 16 | 150 |
|  | 17 | 12.000 | 1.00 | . | . | 17 | 149 |
|  | 18 | 12.000 | 1.00 | . | . | 18 | 148 |
|  | 19 | 12.000 | 1.00 | . | . | 19 | 147 |
|  | 20 | 12.000 | 1.00 | .880 | .025 | 20 | 146 |
|  | 21 | 15.000 | 1.00 | . | . | 21 | 145 |
|  | 22 | 15.000 | 1.00 | . | . | 22 | 144 |
|  | 23 | 15.000 | 1.00 | . | . | 23 | 143 |
|  | 24 | 15.000 | 1.00 | . | . | 24 | 142 |
|  | 25 | 15.000 | 1.00 | . | . | 25 | 141 |
|  | 26 | 15.000 | 1.00 | . | . | 26 | 140 |
|  | 27 | 15.000 | 1.00 | . | . | 27 | 139 |
|  | 28 | 15.000 | 1.00 | .831 | .029 | 28 | 138 |
|  | 29 | 18.000 | 1.00 | . | . | 29 | 137 |
|  | 30 | 18.000 | 1.00 | .819 | .030 | 30 | 136 |
|  | 31 | 21.000 | 1.00 | . | . | 31 | 135 |
|  | 32 | 21.000 | 1.00 | . | . | 32 | 134 |
|  | 33 | 21.000 | 1.00 | . | . | 33 | 133 |
|  | 34 | 21.000 | 1.00 | . | . | 34 | 132 |
|  | 35 | 21.000 | 1.00 | . | . | 35 | 131 |
|  | 36 | 21.000 | 1.00 | . | . | 36 | 130 |
|  | 37 | 21.000 | 1.00 | . | . | 37 | 129 |
|  | 38 | 21.000 | 1.00 | . | . | 38 | 128 |
|  | 39 | 21.000 | 1.00 | . | . | 39 | 127 |
|  | 40 | 21.000 | 1.00 | . | . | 40 | 126 |
|  | 41 | 21.000 | 1.00 | .753 | .033 | 41 | 125 |
|  | 42 | 24.000 | 1.00 | . | . | 42 | 124 |
|  | 43 | 24.000 | 1.00 | . | . | 43 | 123 |
|  | 44 | 24.000 | 1.00 | . | . | 44 | 122 |
|  | 45 | 24.000 | 1.00 | . | . | 45 | 121 |
|  | 46 | 24.000 | 1.00 | . | . | 46 | 120 |
|  | 47 | 24.000 | 1.00 | . | . | 47 | 119 |
|  | 48 | 24.000 | 1.00 | . | . | 48 | 118 |
|  | 49 | 24.000 | 1.00 | . | . | 49 | 117 |
|  | 50 | 24.000 | 1.00 | . | . | 50 | 116 |
|  | 51 | 24.000 | 1.00 | . | . | 51 | 115 |
|  | 52 | 24.000 | 1.00 | . | . | 52 | 114 |
|  | 53 | 24.000 | 1.00 | . | . | 53 | 113 |
|  | 54 | 24.000 | 1.00 | . | . | 54 | 112 |
|  | 55 | 24.000 | 1.00 | . | . | 55 | 111 |
|  | 56 | 24.000 | 1.00 | . | . | 56 | 110 |
|  | 57 | 24.000 | 1.00 | . | . | 57 | 109 |
|  | 58 | 24.000 | 1.00 | . | . | 58 | 108 |
|  | 59 | 24.000 | 1.00 | . | . | 59 | 107 |
|  | 60 | 24.000 | 1.00 | . | . | 60 | 106 |
|  | 61 | 24.000 | 1.00 | . | . | 61 | 105 |
|  | 62 | 24.000 | 1.00 | . | . | 62 | 104 |
|  | 63 | 24.000 | 1.00 | .620 | .038 | 63 | 103 |
|  | 64 | 27.000 | 1.00 | . | . | 64 | 102 |
|  | 65 | 27.000 | 1.00 | . | . | 65 | 101 |
|  | 66 | 27.000 | 1.00 | . | . | 66 | 100 |
|  | 67 | 27.000 | 1.00 | . | . | 67 | 99 |
|  | 68 | 27.000 | 1.00 | .590 | .038 | 68 | 98 |
|  | 69 | 31.000 | 1.00 | . | . | 69 | 97 |
|  | 70 | 31.000 | 1.00 | . | . | 70 | 96 |
|  | 71 | 31.000 | 1.00 | . | . | 71 | 95 |
|  | 72 | 31.000 | 1.00 | . | . | 72 | 94 |
|  | 73 | 31.000 | 1.00 | . | . | 73 | 93 |
|  | 74 | 31.000 | 1.00 | . | . | 74 | 92 |
|  | 75 | 31.000 | 1.00 | . | . | 75 | 91 |
|  | 76 | 31.000 | 1.00 | . | . | 76 | 90 |
|  | 77 | 31.000 | 1.00 | . | . | 77 | 89 |
|  | 78 | 31.000 | 1.00 | . | . | 78 | 88 |
|  | 79 | 31.000 | 1.00 | . | . | 79 | 87 |
|  | 80 | 31.000 | 1.00 | . | . | 80 | 86 |
|  | 81 | 31.000 | 1.00 | . | . | 81 | 85 |
|  | 82 | 31.000 | 1.00 | . | . | 82 | 84 |
|  | 83 | 31.000 | 1.00 | . | . | 83 | 83 |
|  | 84 | 31.000 | 1.00 | . | . | 84 | 82 |
|  | 85 | 31.000 | 1.00 | . | . | 85 | 81 |
|  | 86 | 31.000 | 1.00 | . | . | 86 | 80 |
|  | 87 | 31.000 | 1.00 | . | . | 87 | 79 |
|  | 88 | 31.000 | 1.00 | . | . | 88 | 78 |
|  | 89 | 31.000 | 1.00 | . | . | 89 | 77 |
|  | 90 | 31.000 | 1.00 | . | . | 90 | 76 |
|  | 91 | 31.000 | 1.00 | . | . | 91 | 75 |
|  | 92 | 31.000 | 1.00 | . | . | 92 | 74 |
|  | 93 | 31.000 | 1.00 | . | . | 93 | 73 |
|  | 94 | 31.000 | 1.00 | . | . | 94 | 72 |
|  | 95 | 31.000 | 1.00 | . | . | 95 | 71 |
|  | 96 | 31.000 | 1.00 | . | . | 96 | 70 |
|  | 97 | 31.000 | 1.00 | . | . | 97 | 69 |
|  | 98 | 31.000 | 1.00 | . | . | 98 | 68 |
|  | 99 | 31.000 | 1.00 | . | . | 99 | 67 |
|  | 100 | 31.000 | 1.00 | . | . | 100 | 66 |
|  | 101 | 31.000 | 1.00 | . | . | 101 | 65 |
|  | 102 | 31.000 | 1.00 | . | . | 102 | 64 |
|  | 103 | 31.000 | 1.00 | . | . | 103 | 63 |
|  | 104 | 31.000 | 1.00 | . | . | 104 | 62 |
|  | 105 | 31.000 | 1.00 | .367 | .037 | 105 | 61 |
|  | 106 | 34.000 | 1.00 | . | . | 106 | 60 |
|  | 107 | 34.000 | 1.00 | . | . | 107 | 59 |
|  | 108 | 34.000 | 1.00 | . | . | 108 | 58 |
|  | 109 | 34.000 | 1.00 | . | . | 109 | 57 |
|  | 110 | 34.000 | 1.00 | . | . | 110 | 56 |
|  | 111 | 34.000 | 1.00 | . | . | 111 | 55 |
|  | 112 | 34.000 | 1.00 | . | . | 112 | 54 |
|  | 113 | 34.000 | 1.00 | . | . | 113 | 53 |
|  | 114 | 34.000 | 1.00 | . | . | 114 | 52 |
|  | 115 | 34.000 | 1.00 | . | . | 115 | 51 |
|  | 116 | 34.000 | 1.00 | . | . | 116 | 50 |
|  | 117 | 34.000 | 1.00 | . | . | 117 | 49 |
|  | 118 | 34.000 | 1.00 | . | . | 118 | 48 |
|  | 119 | 34.000 | 1.00 | . | . | 119 | 47 |
|  | 120 | 34.000 | 1.00 | . | . | 120 | 46 |
|  | 121 | 34.000 | 1.00 | . | . | 121 | 45 |
|  | 122 | 34.000 | 1.00 | . | . | 122 | 44 |
|  | 123 | 34.000 | 1.00 | . | . | 123 | 43 |
|  | 124 | 34.000 | 1.00 | . | . | 124 | 42 |
|  | 125 | 34.000 | 1.00 | . | . | 125 | 41 |
|  | 126 | 34.000 | 1.00 | . | . | 126 | 40 |
|  | 127 | 34.000 | 1.00 | . | . | 127 | 39 |
|  | 128 | 34.000 | 1.00 | . | . | 128 | 38 |
|  | 129 | 34.000 | 1.00 | . | . | 129 | 37 |
|  | 130 | 34.000 | 1.00 | . | . | 130 | 36 |
|  | 131 | 34.000 | 1.00 | . | . | 131 | 35 |
|  | 132 | 34.000 | 1.00 | . | . | 132 | 34 |
|  | 133 | 34.000 | 1.00 | . | . | 133 | 33 |
|  | 134 | 34.000 | 1.00 | . | . | 134 | 32 |
|  | 135 | 34.000 | 1.00 | . | . | 135 | 31 |
|  | 136 | 34.000 | 1.00 | . | . | 136 | 30 |
|  | 137 | 34.000 | 1.00 | .175 | .029 | 137 | 29 |
|  | 138 | 37.000 | 1.00 | . | . | 138 | 28 |
|  | 139 | 37.000 | 1.00 | . | . | 139 | 27 |
|  | 140 | 37.000 | 1.00 | . | . | 140 | 26 |
|  | 141 | 37.000 | 1.00 | . | . | 141 | 25 |
|  | 142 | 37.000 | 1.00 | . | . | 142 | 24 |
|  | 143 | 37.000 | 1.00 | . | . | 143 | 23 |
|  | 144 | 37.000 | 1.00 | . | . | 144 | 22 |
|  | 145 | 37.000 | 1.00 | .127 | .026 | 145 | 21 |
|  | 146 | 41.000 | 1.00 | . | . | 146 | 20 |
|  | 147 | 41.000 | 1.00 | . | . | 147 | 19 |
|  | 148 | 41.000 | 1.00 | . | . | 148 | 18 |
|  | 149 | 41.000 | 1.00 | . | . | 149 | 17 |
|  | 150 | 41.000 | 1.00 | . | . | 150 | 16 |
|  | 151 | 41.000 | 1.00 | . | . | 151 | 15 |
|  | 152 | 41.000 | 1.00 | . | . | 152 | 14 |
|  | 153 | 41.000 | 1.00 | . | . | 153 | 13 |
|  | 154 | 41.000 | 1.00 | . | . | 154 | 12 |
|  | 155 | 41.000 | 1.00 | . | . | 155 | 11 |
|  | 156 | 41.000 | 1.00 | . | . | 156 | 10 |
|  | 157 | 41.000 | 1.00 | . | . | 157 | 9 |
|  | 158 | 41.000 | 1.00 | .048 | .017 | 158 | 8 |
|  | 159 | 44.000 | 1.00 | . | . | 159 | 7 |
|  | 160 | 44.000 | 1.00 | .036 | .014 | 160 | 6 |
|  | 161 | 47.000 | 1.00 | . | . | 161 | 5 |
|  | 162 | 47.000 | 1.00 | . | . | 162 | 4 |
|  | 163 | 47.000 | 1.00 | . | . | 163 | 3 |
|  | 164 | 47.000 | 1.00 | .012 | .008 | 164 | 2 |
|  | 165 | 50.000 | 1.00 | . | . | 165 | 1 |
|  | 166 | 50.000 | 1.00 | .000 | .000 | 166 | 0 |
| 3.00 | 1 | 9.000 | 1.00 | . | . | 1 | 151 |
|  | 2 | 9.000 | 1.00 | . | . | 2 | 150 |
|  | 3 | 9.000 | 1.00 | . | . | 3 | 149 |
|  | 4 | 9.000 | 1.00 | .974 | .013 | 4 | 148 |
|  | 5 | 12.000 | 1.00 | . | . | 5 | 147 |
|  | 6 | 12.000 | 1.00 | . | . | 6 | 146 |
|  | 7 | 12.000 | 1.00 | .954 | .017 | 7 | 145 |
|  | 8 | 15.000 | 1.00 | . | . | 8 | 144 |
|  | 9 | 15.000 | 1.00 | .941 | .019 | 9 | 143 |
|  | 10 | 18.000 | 1.00 | . | . | 10 | 142 |
|  | 11 | 18.000 | 1.00 | . | . | 11 | 141 |
|  | 12 | 18.000 | 1.00 | . | . | 12 | 140 |
|  | 13 | 18.000 | 1.00 | .914 | .023 | 13 | 139 |
|  | 14 | 21.000 | 1.00 | . | . | 14 | 138 |
|  | 15 | 21.000 | 1.00 | . | . | 15 | 137 |
|  | 16 | 21.000 | 1.00 | . | . | 16 | 136 |
|  | 17 | 21.000 | 1.00 | . | . | 17 | 135 |
|  | 18 | 21.000 | 1.00 | . | . | 18 | 134 |
|  | 19 | 21.000 | 1.00 | . | . | 19 | 133 |
|  | 20 | 21.000 | 1.00 | . | . | 20 | 132 |
|  | 21 | 21.000 | 1.00 | . | . | 21 | 131 |
|  | 22 | 21.000 | 1.00 | .855 | .029 | 22 | 130 |
|  | 23 | 24.000 | 1.00 | . | . | 23 | 129 |
|  | 24 | 24.000 | 1.00 | . | . | 24 | 128 |
|  | 25 | 24.000 | 1.00 | . | . | 25 | 127 |
|  | 26 | 24.000 | 1.00 | . | . | 26 | 126 |
|  | 27 | 24.000 | 1.00 | . | . | 27 | 125 |
|  | 28 | 24.000 | 1.00 | . | . | 28 | 124 |
|  | 29 | 24.000 | 1.00 | . | . | 29 | 123 |
|  | 30 | 24.000 | 1.00 | . | . | 30 | 122 |
|  | 31 | 24.000 | 1.00 | . | . | 31 | 121 |
|  | 32 | 24.000 | 1.00 | . | . | 32 | 120 |
|  | 33 | 24.000 | 1.00 | . | . | 33 | 119 |
|  | 34 | 24.000 | 1.00 | . | . | 34 | 118 |
|  | 35 | 24.000 | 1.00 | . | . | 35 | 117 |
|  | 36 | 24.000 | 1.00 | . | . | 36 | 116 |
|  | 37 | 24.000 | 1.00 | . | . | 37 | 115 |
|  | 38 | 24.000 | 1.00 | . | . | 38 | 114 |
|  | 39 | 24.000 | 1.00 | . | . | 39 | 113 |
|  | 40 | 24.000 | 1.00 | . | . | 40 | 112 |
|  | 41 | 24.000 | 1.00 | . | . | 41 | 111 |
|  | 42 | 24.000 | 1.00 | . | . | 42 | 110 |
|  | 43 | 24.000 | 1.00 | . | . | 43 | 109 |
|  | 44 | 24.000 | 1.00 | . | . | 44 | 108 |
|  | 45 | 24.000 | 1.00 | . | . | 45 | 107 |
|  | 46 | 24.000 | 1.00 | . | . | 46 | 106 |
|  | 47 | 24.000 | 1.00 | . | . | 47 | 105 |
|  | 48 | 24.000 | 1.00 | . | . | 48 | 104 |
|  | 49 | 24.000 | 1.00 | . | . | 49 | 103 |
|  | 50 | 24.000 | 1.00 | . | . | 50 | 102 |
|  | 51 | 24.000 | 1.00 | . | . | 51 | 101 |
|  | 52 | 24.000 | 1.00 | . | . | 52 | 100 |
|  | 53 | 24.000 | 1.00 | . | . | 53 | 99 |
|  | 54 | 24.000 | 1.00 | . | . | 54 | 98 |
|  | 55 | 24.000 | 1.00 | . | . | 55 | 97 |
|  | 56 | 24.000 | 1.00 | . | . | 56 | 96 |
|  | 57 | 24.000 | 1.00 | . | . | 57 | 95 |
|  | 58 | 24.000 | 1.00 | . | . | 58 | 94 |
|  | 59 | 24.000 | 1.00 | . | . | 59 | 93 |
|  | 60 | 24.000 | 1.00 | . | . | 60 | 92 |
|  | 61 | 24.000 | 1.00 | . | . | 61 | 91 |
|  | 62 | 24.000 | 1.00 | . | . | 62 | 90 |
|  | 63 | 24.000 | 1.00 | . | . | 63 | 89 |
|  | 64 | 24.000 | 1.00 | . | . | 64 | 88 |
|  | 65 | 24.000 | 1.00 | . | . | 65 | 87 |
|  | 66 | 24.000 | 1.00 | . | . | 66 | 86 |
|  | 67 | 24.000 | 1.00 | . | . | 67 | 85 |
|  | 68 | 24.000 | 1.00 | . | . | 68 | 84 |
|  | 69 | 24.000 | 1.00 | . | . | 69 | 83 |
|  | 70 | 24.000 | 1.00 | . | . | 70 | 82 |
|  | 71 | 24.000 | 1.00 | . | . | 71 | 81 |
|  | 72 | 24.000 | 1.00 | . | . | 72 | 80 |
|  | 73 | 24.000 | 1.00 | . | . | 73 | 79 |
|  | 74 | 24.000 | 1.00 | .513 | .041 | 74 | 78 |
|  | 75 | 27.000 | 1.00 | . | . | 75 | 77 |
|  | 76 | 27.000 | 1.00 | . | . | 76 | 76 |
|  | 77 | 27.000 | 1.00 | . | . | 77 | 75 |
|  | 78 | 27.000 | 1.00 | . | . | 78 | 74 |
|  | 79 | 27.000 | 1.00 | . | . | 79 | 73 |
|  | 80 | 27.000 | 1.00 | . | . | 80 | 72 |
|  | 81 | 27.000 | 1.00 | . | . | 81 | 71 |
|  | 82 | 27.000 | 1.00 | . | . | 82 | 70 |
|  | 83 | 27.000 | 1.00 | . | . | 83 | 69 |
|  | 84 | 27.000 | 1.00 | . | . | 84 | 68 |
|  | 85 | 27.000 | 1.00 | . | . | 85 | 67 |
|  | 86 | 27.000 | 1.00 | . | . | 86 | 66 |
|  | 87 | 27.000 | 1.00 | . | . | 87 | 65 |
|  | 88 | 27.000 | 1.00 | . | . | 88 | 64 |
|  | 89 | 27.000 | 1.00 | . | . | 89 | 63 |
|  | 90 | 27.000 | 1.00 | .408 | .040 | 90 | 62 |
|  | 91 | 31.000 | 1.00 | . | . | 91 | 61 |
|  | 92 | 31.000 | 1.00 | . | . | 92 | 60 |
|  | 93 | 31.000 | 1.00 | . | . | 93 | 59 |
|  | 94 | 31.000 | 1.00 | . | . | 94 | 58 |
|  | 95 | 31.000 | 1.00 | . | . | 95 | 57 |
|  | 96 | 31.000 | 1.00 | . | . | 96 | 56 |
|  | 97 | 31.000 | 1.00 | . | . | 97 | 55 |
|  | 98 | 31.000 | 1.00 | . | . | 98 | 54 |
|  | 99 | 31.000 | 1.00 | . | . | 99 | 53 |
|  | 100 | 31.000 | 1.00 | . | . | 100 | 52 |
|  | 101 | 31.000 | 1.00 | . | . | 101 | 51 |
|  | 102 | 31.000 | 1.00 | . | . | 102 | 50 |
|  | 103 | 31.000 | 1.00 | . | . | 103 | 49 |
|  | 104 | 31.000 | 1.00 | . | . | 104 | 48 |
|  | 105 | 31.000 | 1.00 | . | . | 105 | 47 |
|  | 106 | 31.000 | 1.00 | . | . | 106 | 46 |
|  | 107 | 31.000 | 1.00 | . | . | 107 | 45 |
|  | 108 | 31.000 | 1.00 | . | . | 108 | 44 |
|  | 109 | 31.000 | 1.00 | . | . | 109 | 43 |
|  | 110 | 31.000 | 1.00 | . | . | 110 | 42 |
|  | 111 | 31.000 | 1.00 | . | . | 111 | 41 |
|  | 112 | 31.000 | 1.00 | . | . | 112 | 40 |
|  | 113 | 31.000 | 1.00 | . | . | 113 | 39 |
|  | 114 | 31.000 | 1.00 | . | . | 114 | 38 |
|  | 115 | 31.000 | 1.00 | . | . | 115 | 37 |
|  | 116 | 31.000 | 1.00 | . | . | 116 | 36 |
|  | 117 | 31.000 | 1.00 | . | . | 117 | 35 |
|  | 118 | 31.000 | 1.00 | . | . | 118 | 34 |
|  | 119 | 31.000 | 1.00 | . | . | 119 | 33 |
|  | 120 | 31.000 | 1.00 | . | . | 120 | 32 |
|  | 121 | 31.000 | 1.00 | . | . | 121 | 31 |
|  | 122 | 31.000 | 1.00 | . | . | 122 | 30 |
|  | 123 | 31.000 | 1.00 | . | . | 123 | 29 |
|  | 124 | 31.000 | 1.00 | .184 | .031 | 124 | 28 |
|  | 125 | 34.000 | 1.00 | . | . | 125 | 27 |
|  | 126 | 34.000 | 1.00 | . | . | 126 | 26 |
|  | 127 | 34.000 | 1.00 | . | . | 127 | 25 |
|  | 128 | 34.000 | 1.00 | . | . | 128 | 24 |
|  | 129 | 34.000 | 1.00 | . | . | 129 | 23 |
|  | 130 | 34.000 | 1.00 | . | . | 130 | 22 |
|  | 131 | 34.000 | 1.00 | . | . | 131 | 21 |
|  | 132 | 34.000 | 1.00 | . | . | 132 | 20 |
|  | 133 | 34.000 | 1.00 | . | . | 133 | 19 |
|  | 134 | 34.000 | 1.00 | . | . | 134 | 18 |
|  | 135 | 34.000 | 1.00 | . | . | 135 | 17 |
|  | 136 | 34.000 | 1.00 | . | . | 136 | 16 |
|  | 137 | 34.000 | 1.00 | . | . | 137 | 15 |
|  | 138 | 34.000 | 1.00 | . | . | 138 | 14 |
|  | 139 | 34.000 | 1.00 | .086 | .023 | 139 | 13 |
|  | 140 | 37.000 | 1.00 | . | . | 140 | 12 |
|  | 141 | 37.000 | 1.00 | . | . | 141 | 11 |
|  | 142 | 37.000 | 1.00 | . | . | 142 | 10 |
|  | 143 | 37.000 | 1.00 | . | . | 143 | 9 |
|  | 144 | 37.000 | 1.00 | . | . | 144 | 8 |
|  | 145 | 37.000 | 1.00 | . | . | 145 | 7 |
|  | 146 | 37.000 | 1.00 | . | . | 146 | 6 |
|  | 147 | 37.000 | 1.00 | .033 | .014 | 147 | 5 |
|  | 148 | 41.000 | 1.00 | . | . | 148 | 4 |
|  | 149 | 41.000 | 1.00 | . | . | 149 | 3 |
|  | 150 | 41.000 | 1.00 | .013 | .009 | 150 | 2 |
|  | 151 | 44.000 | 1.00 | . | . | 151 | 1 |
|  | 152 | 44.000 | 1.00 | .000 | .000 | 152 | 0 |
| 4.00 | 1 | 6.000 | 1.00 | .993 | .007 | 1 | 148 |
|  | 2 | 9.000 | 1.00 | . | . | 2 | 147 |
|  | 3 | 9.000 | 1.00 | . | . | 3 | 146 |
|  | 4 | 9.000 | 1.00 | .973 | .013 | 4 | 145 |
|  | 5 | 12.000 | 1.00 | . | . | 5 | 144 |
|  | 6 | 12.000 | 1.00 | . | . | 6 | 143 |
|  | 7 | 12.000 | 1.00 | . | . | 7 | 142 |
|  | 8 | 12.000 | 1.00 | . | . | 8 | 141 |
|  | 9 | 12.000 | 1.00 | . | . | 9 | 140 |
|  | 10 | 12.000 | 1.00 | .933 | .020 | 10 | 139 |
|  | 11 | 15.000 | 1.00 | . | . | 11 | 138 |
|  | 12 | 15.000 | 1.00 | . | . | 12 | 137 |
|  | 13 | 15.000 | 1.00 | . | . | 13 | 136 |
|  | 14 | 15.000 | 1.00 | .906 | .024 | 14 | 135 |
|  | 15 | 18.000 | 1.00 | .899 | .025 | 15 | 134 |
|  | 16 | 21.000 | 1.00 | . | . | 16 | 133 |
|  | 17 | 21.000 | 1.00 | . | . | 17 | 132 |
|  | 18 | 21.000 | 1.00 | . | . | 18 | 131 |
|  | 19 | 21.000 | 1.00 | . | . | 19 | 130 |
|  | 20 | 21.000 | 1.00 | . | . | 20 | 129 |
|  | 21 | 21.000 | 1.00 | . | . | 21 | 128 |
|  | 22 | 21.000 | 1.00 | . | . | 22 | 127 |
|  | 23 | 21.000 | 1.00 | . | . | 23 | 126 |
|  | 24 | 21.000 | 1.00 | . | . | 24 | 125 |
|  | 25 | 21.000 | 1.00 | .832 | .031 | 25 | 124 |
|  | 26 | 24.000 | 1.00 | . | . | 26 | 123 |
|  | 27 | 24.000 | 1.00 | . | . | 27 | 122 |
|  | 28 | 24.000 | 1.00 | . | . | 28 | 121 |
|  | 29 | 24.000 | 1.00 | . | . | 29 | 120 |
|  | 30 | 24.000 | 1.00 | . | . | 30 | 119 |
|  | 31 | 24.000 | 1.00 | . | . | 31 | 118 |
|  | 32 | 24.000 | 1.00 | . | . | 32 | 117 |
|  | 33 | 24.000 | 1.00 | . | . | 33 | 116 |
|  | 34 | 24.000 | 1.00 | . | . | 34 | 115 |
|  | 35 | 24.000 | 1.00 | . | . | 35 | 114 |
|  | 36 | 24.000 | 1.00 | . | . | 36 | 113 |
|  | 37 | 24.000 | 1.00 | . | . | 37 | 112 |
|  | 38 | 24.000 | 1.00 | . | . | 38 | 111 |
|  | 39 | 24.000 | 1.00 | . | . | 39 | 110 |
|  | 40 | 24.000 | 1.00 | . | . | 40 | 109 |
|  | 41 | 24.000 | 1.00 | . | . | 41 | 108 |
|  | 42 | 24.000 | 1.00 | . | . | 42 | 107 |
|  | 43 | 24.000 | 1.00 | . | . | 43 | 106 |
|  | 44 | 24.000 | 1.00 | . | . | 44 | 105 |
|  | 45 | 24.000 | 1.00 | . | . | 45 | 104 |
|  | 46 | 24.000 | 1.00 | . | . | 46 | 103 |
|  | 47 | 24.000 | 1.00 | . | . | 47 | 102 |
|  | 48 | 24.000 | 1.00 | . | . | 48 | 101 |
|  | 49 | 24.000 | 1.00 | . | . | 49 | 100 |
|  | 50 | 24.000 | 1.00 | . | . | 50 | 99 |
|  | 51 | 24.000 | 1.00 | . | . | 51 | 98 |
|  | 52 | 24.000 | 1.00 | . | . | 52 | 97 |
|  | 53 | 24.000 | 1.00 | . | . | 53 | 96 |
|  | 54 | 24.000 | 1.00 | . | . | 54 | 95 |
|  | 55 | 24.000 | 1.00 | . | . | 55 | 94 |
|  | 56 | 24.000 | 1.00 | . | . | 56 | 93 |
|  | 57 | 24.000 | 1.00 | . | . | 57 | 92 |
|  | 58 | 24.000 | 1.00 | . | . | 58 | 91 |
|  | 59 | 24.000 | 1.00 | . | . | 59 | 90 |
|  | 60 | 24.000 | 1.00 | . | . | 60 | 89 |
|  | 61 | 24.000 | 1.00 | .591 | .040 | 61 | 88 |
|  | 62 | 27.000 | 1.00 | . | . | 62 | 87 |
|  | 63 | 27.000 | 1.00 | . | . | 63 | 86 |
|  | 64 | 27.000 | 1.00 | .570 | .041 | 64 | 85 |
|  | 65 | 31.000 | 1.00 | . | . | 65 | 84 |
|  | 66 | 31.000 | 1.00 | . | . | 66 | 83 |
|  | 67 | 31.000 | 1.00 | . | . | 67 | 82 |
|  | 68 | 31.000 | 1.00 | . | . | 68 | 81 |
|  | 69 | 31.000 | 1.00 | . | . | 69 | 80 |
|  | 70 | 31.000 | 1.00 | . | . | 70 | 79 |
|  | 71 | 31.000 | 1.00 | . | . | 71 | 78 |
|  | 72 | 31.000 | 1.00 | . | . | 72 | 77 |
|  | 73 | 31.000 | 1.00 | . | . | 73 | 76 |
|  | 74 | 31.000 | 1.00 | . | . | 74 | 75 |
|  | 75 | 31.000 | 1.00 | . | . | 75 | 74 |
|  | 76 | 31.000 | 1.00 | . | . | 76 | 73 |
|  | 77 | 31.000 | 1.00 | . | . | 77 | 72 |
|  | 78 | 31.000 | 1.00 | . | . | 78 | 71 |
|  | 79 | 31.000 | 1.00 | . | . | 79 | 70 |
|  | 80 | 31.000 | 1.00 | . | . | 80 | 69 |
|  | 81 | 31.000 | 1.00 | . | . | 81 | 68 |
|  | 82 | 31.000 | 1.00 | . | . | 82 | 67 |
|  | 83 | 31.000 | 1.00 | . | . | 83 | 66 |
|  | 84 | 31.000 | 1.00 | . | . | 84 | 65 |
|  | 85 | 31.000 | 1.00 | . | . | 85 | 64 |
|  | 86 | 31.000 | 1.00 | . | . | 86 | 63 |
|  | 87 | 31.000 | 1.00 | . | . | 87 | 62 |
|  | 88 | 31.000 | 1.00 | .409 | .040 | 88 | 61 |
|  | 89 | 34.000 | 1.00 | . | . | 89 | 60 |
|  | 90 | 34.000 | 1.00 | . | . | 90 | 59 |
|  | 91 | 34.000 | 1.00 | . | . | 91 | 58 |
|  | 92 | 34.000 | 1.00 | . | . | 92 | 57 |
|  | 93 | 34.000 | 1.00 | . | . | 93 | 56 |
|  | 94 | 34.000 | 1.00 | . | . | 94 | 55 |
|  | 95 | 34.000 | 1.00 | . | . | 95 | 54 |
|  | 96 | 34.000 | 1.00 | . | . | 96 | 53 |
|  | 97 | 34.000 | 1.00 | . | . | 97 | 52 |
|  | 98 | 34.000 | 1.00 | . | . | 98 | 51 |
|  | 99 | 34.000 | 1.00 | . | . | 99 | 50 |
|  | 100 | 34.000 | 1.00 | . | . | 100 | 49 |
|  | 101 | 34.000 | 1.00 | . | . | 101 | 48 |
|  | 102 | 34.000 | 1.00 | . | . | 102 | 47 |
|  | 103 | 34.000 | 1.00 | . | . | 103 | 46 |
|  | 104 | 34.000 | 1.00 | . | . | 104 | 45 |
|  | 105 | 34.000 | 1.00 | . | . | 105 | 44 |
|  | 106 | 34.000 | 1.00 | . | . | 106 | 43 |
|  | 107 | 34.000 | 1.00 | . | . | 107 | 42 |
|  | 108 | 34.000 | 1.00 | . | . | 108 | 41 |
|  | 109 | 34.000 | 1.00 | . | . | 109 | 40 |
|  | 110 | 34.000 | 1.00 | . | . | 110 | 39 |
|  | 111 | 34.000 | 1.00 | . | . | 111 | 38 |
|  | 112 | 34.000 | 1.00 | . | . | 112 | 37 |
|  | 113 | 34.000 | 1.00 | .242 | .035 | 113 | 36 |
|  | 114 | 37.000 | 1.00 | . | . | 114 | 35 |
|  | 115 | 37.000 | 1.00 | . | . | 115 | 34 |
|  | 116 | 37.000 | 1.00 | . | . | 116 | 33 |
|  | 117 | 37.000 | 1.00 | . | . | 117 | 32 |
|  | 118 | 37.000 | 1.00 | . | . | 118 | 31 |
|  | 119 | 37.000 | 1.00 | . | . | 119 | 30 |
|  | 120 | 37.000 | 1.00 | . | . | 120 | 29 |
|  | 121 | 37.000 | 1.00 | . | . | 121 | 28 |
|  | 122 | 37.000 | 1.00 | . | . | 122 | 27 |
|  | 123 | 37.000 | 1.00 | . | . | 123 | 26 |
|  | 124 | 37.000 | 1.00 | . | . | 124 | 25 |
|  | 125 | 37.000 | 1.00 | . | . | 125 | 24 |
|  | 126 | 37.000 | 1.00 | . | . | 126 | 23 |
|  | 127 | 37.000 | 1.00 | . | . | 127 | 22 |
|  | 128 | 37.000 | 1.00 | . | . | 128 | 21 |
|  | 129 | 37.000 | 1.00 | . | . | 129 | 20 |
|  | 130 | 37.000 | 1.00 | . | . | 130 | 19 |
|  | 131 | 37.000 | 1.00 | . | . | 131 | 18 |
|  | 132 | 37.000 | 1.00 | .114 | .026 | 132 | 17 |
|  | 133 | 41.000 | 1.00 | . | . | 133 | 16 |
|  | 134 | 41.000 | 1.00 | . | . | 134 | 15 |
|  | 135 | 41.000 | 1.00 | . | . | 135 | 14 |
|  | 136 | 41.000 | 1.00 | . | . | 136 | 13 |
|  | 137 | 41.000 | 1.00 | . | . | 137 | 12 |
|  | 138 | 41.000 | 1.00 | . | . | 138 | 11 |
|  | 139 | 41.000 | 1.00 | .067 | .020 | 139 | 10 |
|  | 140 | 44.000 | 1.00 | . | . | 140 | 9 |
|  | 141 | 44.000 | 1.00 | . | . | 141 | 8 |
|  | 142 | 44.000 | 1.00 | . | . | 142 | 7 |
|  | 143 | 44.000 | 1.00 | . | . | 143 | 6 |
|  | 144 | 44.000 | 1.00 | .034 | .015 | 144 | 5 |
|  | 145 | 47.000 | 1.00 | . | . | 145 | 4 |
|  | 146 | 47.000 | 1.00 | . | . | 146 | 3 |
|  | 147 | 47.000 | 1.00 | . | . | 147 | 2 |
|  | 148 | 47.000 | 1.00 | .007 | .007 | 148 | 1 |
|  | 149 | 50.000 | 1.00 | .000 | .000 | 149 | 0 |

| **Means and Medians for Survival Time** | | | | | | |
| --- | --- | --- | --- | --- | --- | --- |
| group | Mean^a^ | | | | Median | |
|  |  | | 95% Confidence Interval | |  | |
|  | Estimate | Std. Error | Lower Bound | Upper Bound | Estimate | Std. Error |
| 1.00 | 26.270 | .720 | 24.859 | 27.682 | 27.000 | .923 |
| 2.00 | 28.205 | .777 | 26.682 | 29.728 | 31.000 | .672 |
| 3.00 | 27.066 | .537 | 26.013 | 28.118 | 27.000 | .535 |
| 4.00 | 29.396 | .729 | 27.968 | 30.824 | 31.000 | 1.000 |
| Overall | 27.746 | .353 | 27.055 | 28.438 | 31.000 | .484 |
| a. Estimation is limited to the largest survival time if it is censored. | | | | | | |

| **Means and Medians for Survival Time** | | |
| --- | --- | --- |
| group | Median | |
|  | 95% Confidence Interval | |
|  | Lower Bound | Upper Bound |
| 1.00 | 25.192 | 28.808 |
| 2.00 | 29.684 | 32.316 |
| 3.00 | 25.952 | 28.048 |
| 4.00 | 29.039 | 32.961 |
| Overall | 30.051 | 31.949 |
|  | | |

| **Pairwise Comparisons** | | | | |
| --- | --- | --- | --- | --- |
|  | group | 1.00 | | 2.00 |
|  |  | Chi-Square | Sig. | Chi-Square |
| Log Rank (Mantel-Cox) | 1.00 |  |  | 7.037 |
|  | 2.00 | 7.037 | .008 |  |
|  | 3.00 | .131 | .717 | 10.316 |
|  | 4.00 | 11.313 | .001 | .454 |

| **Pairwise Comparisons** | | | | | | |
| --- | --- | --- | --- | --- | --- | --- |
|  | group | 2.00 | 3.00 | | 4.00 | |
|  |  | Sig. | Chi-Square | Sig. | Chi-Square | Sig. |
| Log Rank (Mantel-Cox) | 1.00 | .008 | .131 | .717 | 11.313 | .001 |
|  | 2.00 |  | 10.316 | .001 | .454 | .501 |
|  | 3.00 | .001 |  |  | 14.846 | .000 |
|  | 4.00 | .501 | 14.846 | .000 |  |  |
