# Supplementary material for: rBmαTX14 Increases the Life Span and Promotes the Locomotion of Caenorhabditis Elegans
Source: PLoS One. 2016 Sep 9;11(9):e0161847. doi: 10.1371/journal.pone.0161847 (PMC5017660; doi:10.1371/journal.pone.0161847)
Supplement: S4 Table — Life span analysis of empty vector, pET28a-rBmαTX14 and pET28a-rBmαTX14 (NEG) were starting from L4. (DOC) [file pone.0161847.s005.doc]

**S4 Table. Effects of pET28a-r*Bmα*TX14 and pET28a-r*Bmα*TX14 (NEG) on longevity. Life span analysis of empty vector, pET28a-r*Bmα*TX14 and pET28a-r*Bmα*TX14 (NEG) were starting from L4.**

| **Strain** | | **Treatment** | **Mean life span±SEM (days)** | | | **Median life span±SEM (days)** | **Maximum life spans (days)** | | ***p*-value vs. control** | **Life span extension** | **Number of animal** | |
| --- | --- | --- | --- | --- | --- | --- | --- | --- | --- | --- | --- | --- |
| N2 | Empty vector | | | 26±0.5 | 27±0.4 | | | 44 | - | - | | 353 |
| N2 | pET28a-r*Bmα*TX14 | | | 28±0.6 | 30±0.7 | | | 51 | 0.000 | 11.1% | | 293 |
| N2 | pET28a-r*Bmα*TX14 (NEG) | | | 27.5±0.5 | 27±0.6 | | | 48 | 0.53 | - | | 301 |

N2 represents the wild type *C. elegans*. SEM=standard error of the mean. *p*-values (log-rank test) refer to the control experiment.
